# A DNA-Binding Protein Tunes Septum Placement during *Bacillus subtilis* Sporulation

**DOI:** 10.1128/JB.00287-19

**Published:** 2019-07-24

**Authors:** Emily E. Brown, Allyssa K. Miller, Inna V. Krieger, Ryan M. Otto, James C. Sacchettini, Jennifer K. Herman

**Affiliations:** a Department of Biochemistry and Biophysics, Texas A&M University, College Station, Texas, USA; b Department of Chemistry, Texas A&M University, College Station, Texas, USA; Ohio State University

**Keywords:** *Bacillus subtilis*, FtsZ, RefZ, nucleoid occlusion, sporulation

## Abstract

Bacillus subtilis is a bacterium capable of differentiating into a spore form more resistant to environmental stress. Early in sporulation, each cell possesses two copies of a circular chromosome. A polar FtsZ ring (Z ring) directs septation over one of the chromosomes, generating two cell compartments. The smaller “forespore” compartment initially contains only 25 to 30% of one chromosome, and this transient genetic asymmetry is required for differentiation. Timely assembly of polar Z rings and precise capture of the chromosome in the forespore both require the DNA-binding protein RefZ. To mediate its role in chromosome capture, RefZ must bind to specific DNA motifs (*RBM*s) that localize near the poles at the time of septation. Cells artificially induced to express RefZ during vegetative growth cannot assemble Z rings, an effect that also requires DNA binding. We hypothesized that RefZ-*RBM* complexes mediate precise chromosome capture by modulating FtsZ function. To investigate, we isolated 10 RefZ loss-of-function (rLOF) variants unable to inhibit cell division yet still capable of binding *RBM*s. Sporulating cells expressing the rLOF variants in place of wild-type RefZ phenocopied a Δ*refZ* mutant, suggesting that RefZ acts through an FtsZ-dependent mechanism. The crystal structure of RefZ was solved, and wild-type RefZ and the rLOF variants were further characterized. Our data suggest that RefZ’s oligomerization state and specificity for the *RBM*s are critical determinants influencing RefZ’s ability to affect FtsZ dynamics. We propose that *RBM*-bound RefZ complexes function as a developmentally regulated nucleoid occlusion system for fine-tuning the position of the septum relative to the chromosome during sporulation.

**IMPORTANCE** The bacterial nucleoid forms a large, highly organized structure. Thus, in addition to storing the genetic code, the nucleoid harbors positional information that can be leveraged by DNA-binding proteins to spatially constrain cellular activities. During B. subtilis sporulation, the nucleoid undergoes reorganization, and the cell division protein FtsZ assembles polarly to direct septation over one chromosome. The TetR family protein RefZ binds DNA motifs (*RBM*s) localized near the poles at the time of division and is required for both timely FtsZ assembly and precise capture of DNA in the future spore compartment. Our data suggest that RefZ exploits nucleoid organization by associating with polarly localized *RBM*s to modulate the positioning of FtsZ relative to the chromosome during sporulation.

## Abstract (precis)

The bacterial nucleoid forms a large, highly organized structure. Thus, in addition to storing the genetic code, the nucleoid harbors positional information that can be leveraged by DNA-binding proteins to spatially constrain cellular activities. During B. subtilis sporulation, the nucleoid undergoes reorganization, and the cell division protein FtsZ assembles polarly to direct septation over one chromosome. The TetR family protein RefZ binds DNA motifs (*RBM*s) localized near the poles at the time of division and is required for both timely FtsZ assembly and precise capture of DNA in the future spore compartment. Our data suggest that RefZ exploits nucleoid organization by associating with polarly localized *RBM*s to modulate the positioning of FtsZ relative to the chromosome during sporulation.

## INTRODUCTION

To regulate cellular processes spatially, some macromolecules within the cell must assume a nonuniform distribution. One way that bacteria create heterogeneity along the bacterial envelope is to utilize proteins that induce and/or partition to sites of membrane curvature (1, 2). From there, membrane curvature proteins can serve as a platform for the localization of additional molecules in the cell. For example, in the rod-shaped bacterium Bacillus subtilis, the negative membrane curvature-sensing protein DivIVA coalesces adjacent to past and future cell division sites, where it then recruits a cell division-inhibitory system called Min to inhibit FtsZ polymerization (3–7). Another mechanism to restrict physiological processes to specific cellular regions is to require that molecules assemble into larger, multisubunit complexes to be active. For example, cell division, which requires the coordinated synthesis and turnover of all layers of the cell envelope, is carried out by a localized multisubunit complex comprised of over 30 proteins called the “divisome” (8).

Like the cell envelope, the highly organized (9) bacterial nucleoid is also utilized to regulate processes spatially. DNA-binding proteins that recognize specific motifs regulate the initiation of DNA replication (10), mediate DNA repair and recombination (11, 12), and segregate chromosomes (13–16). Moreover, some DNA-binding proteins simultaneously interact with the nucleoid and the cell envelope to perform functions in DNA replication (17, 18), chromosome organization (19–22), DNA segregation (23), and regulation of cell division (24).

The most extensively studied example of a DNA-binding protein that regulates cell division is SlmA, a TetR family member found in Escherichia coli (25), as well as several other important *Gammaproteobacteria* (26). E. coli SlmA binds to dozens of motifs (SBSs) distributed throughout the chromosome, except in the terminus (*ter*) region (27, 28). In a mechanism termed nucleoid occlusion (NO), SlmA-SBS complexes inhibit cell division by disrupting polymerization of FtsZ (27, 28). By restricting SlmA activity to sites of SBS enrichment, E. coli effectively inhibits the formation of Z rings over the bulk nucleoid while at the same time permitting Z ring assembly in the midcell-localized *ter* region. In this way, SlmA utilizes the chromosome as a landmark to spatially regulate its FtsZ-inhibitory function.

Like E. coli, B. subtilis also possesses a NO system to prevent cell division over the bulk nucleoid (29, 30). The NO system of B. subtilis is comprised of a DNA-binding protein, Noc, and its cognate binding sites (Noc-binding sites [NBSs]), which are also distributed throughout the chromosome with a notable gap in the *ter* region (30). In contrast to SlmA, evidence for a direct interaction between Noc and FtsZ is currently lacking. Instead, Noc-NBS complexes associate with the cell envelope, where they are hypothesized to perturb the association and/or nucleation of FtsZ filaments at the membrane (24).

During B. subtilis sporulation, several morphological changes occur to facilitate spore formation. The cell’s two chromosomes are stretched from pole to pole in an elongated *oriC-ter-ter-oriC* configuration called the axial filament (31, 32). In addition, there is a dramatic adjustment in the location of cell division, with FtsZ shifting from midcell toward a cell quarter, directing septation over one chromosome. During sporulation, Z ring inhibition imposed by both the Min and NO systems must be relieved. Alleviation of Min inhibition may be facilitated by the repositioning of MinD (required to mediate MinC-dependent inhibition of FtsZ) to the distal cell pole (33). Regarding NO, it has been proposed that the axial filament may be arranged so that relatively few Noc-binding sites are positioned at the site of incipient septation (30).

The shift of FtsZ from midcell toward the pole is promoted by increased levels of FtsZ (34, 35) and expression of a membrane-associated sporulation protein, SpoIIE (36, 37). Following septation, the larger mother cell possesses an entire chromosome, whereas the forespore initially contains only one-quarter to one-third of the second chromosome (14, 32). The genetic asymmetry between the mother cell and forespore is critical for differentiation (38, 39), and the region captured is reproducible (14, 32). The chromosome is not bisected during polar division because SpoIIIE, a DNA translocase localized to the edge of the septum (40), assembles around the chromosomal arms (23, 41). Since the chromosome is threaded through the septum, SpoIIIE must directionally pump the remainder from the mother cell into the forespore for development to progress. To avoid chromosome breakage during septation, capture a reproducible region of DNA in the forespore, and pump the forespore-destined chromosome in the correct direction, there must be coordination between cell division proteins, SpoIIIE, and the chromosome. How this coordination is orchestrated at the molecular level largely remains a mystery.

Precise division over and capture of the forespore-destined chromosome requires RefZ, a TetR family DNA-binding protein conserved across the genus *Bacillus* (42, 43). RefZ expression is activated early in sporulation, first via the stationary-phase sigma factor σH (44) and then by phosphorylated Spo0A (Spo0A∼P), the activated form of the sporulation master response regulator (45, 46). RefZ binds to five nearly palindromic DNA motifs (*RBM*s), two on each chromosomal arm and one near *oriC* (42, 43). The *RBM*s on the left and right arms delineate the boundary between chromosomal regions present in the forespore and mother cell at the time of septation. Chromosomal regions immediately adjacent to each *RBM* localize near the incipient site of polar cell division, suggesting a possible role in division or organization of the chromosome near the sporulation septum (42). Consistent with this idea, the *RBM*s are required for precise capture of the forespore-destined chromosome (42). Strikingly, the relative position of the *RBM*s with respect to *oriC* is conserved across the entire genus *Bacillus*. This evolutionary conservation strongly suggests that the location of the *RBM*s is functionally important and provides a considerable selective advantage to the genus (42).

In addition to imprecise chromosome capture, perturbation of RefZ activity is associated with two other phenotypes: first, during sporulation, a Δ*refZ* mutant is modestly delayed in assembly of polar Z rings (43). Second, artificially induced expression of RefZ during vegetative growth disrupts Z ring assembly and inhibits cell division. RefZ-DNA complexes are likely required to disrupt Z rings, as RefZ DNA-binding mutants no longer disrupt cell division (43). These data, and the fact that RefZ and SlmA are both TetR family proteins, led us to hypothesize that *RBM*-bound RefZ complexes might act as a developmentally regulated NO system that tunes FtsZ dynamics and/or Z ring positioning relative to the chromosome.

To test this hypothesis, we isolated and characterized 10 RefZ loss-of-function (rLOF) variants unable to inhibit cell division when artificially induced during vegetative growth yet still capable of binding *RBM*s. None of the rLOF variants were able to support wild-type (WT) chromosome capture when expressed from the native promoter during sporulation and instead phenocopied a Δ*refZ* mutant. These results are consistent with a model in which RefZ mediates precise chromosome capture by modulating FtsZ activity. To better understand the molecular basis of RefZ’s activity, wild-type RefZ and the rLOF variants were overexpressed and purified, and structural and biochemical characterizations were carried out. The locations of the rLOF substitutions on the RefZ crystal structure suggest that RefZ affects FtsZ through a mechanism that is distinct from that described for SlmA. Characterization of the rLOF variants indicates that specificity for *RBM*-containing DNA and RefZ’s propensity to dimerize are critical determinants governing RefZ’s effect on cell division and precise capture of forespore chromosomes *in vivo*.

## RESULTS

### Identification of RefZ residues important for inhibition of cell division.

Artificial expression of RefZ during vegetative growth disrupts Z ring formation and inhibits cell division, resulting in filamentation (43). The division inhibition phenotype can be suppressed in strain backgrounds harboring specific mutations in *ftsZ* or a second copy of the *ftsAZ* operon (43). Division inhibition appears to require RefZ’s DNA-binding activity, as RefZ variants harboring substitutions in the DNA recognition helix (Y43A and Y44A) do not filament cells following artificial expression (43). DNA binding is also likely required for RefZ’s role in chromosome capture, as a strain harboring point mutations in the five *oriC*-proximal RefZ binding motifs (*RBM_5mu_*) exhibits the same capture defect as a Δ*refZ* mutant (42). Based on these data, we hypothesized that RefZ associates with *RBM*s to modulate FtsZ dynamics in the vicinity of the incipient septum and that this modulation would be required to ensure precise chromosome capture.

**FIG 1:**
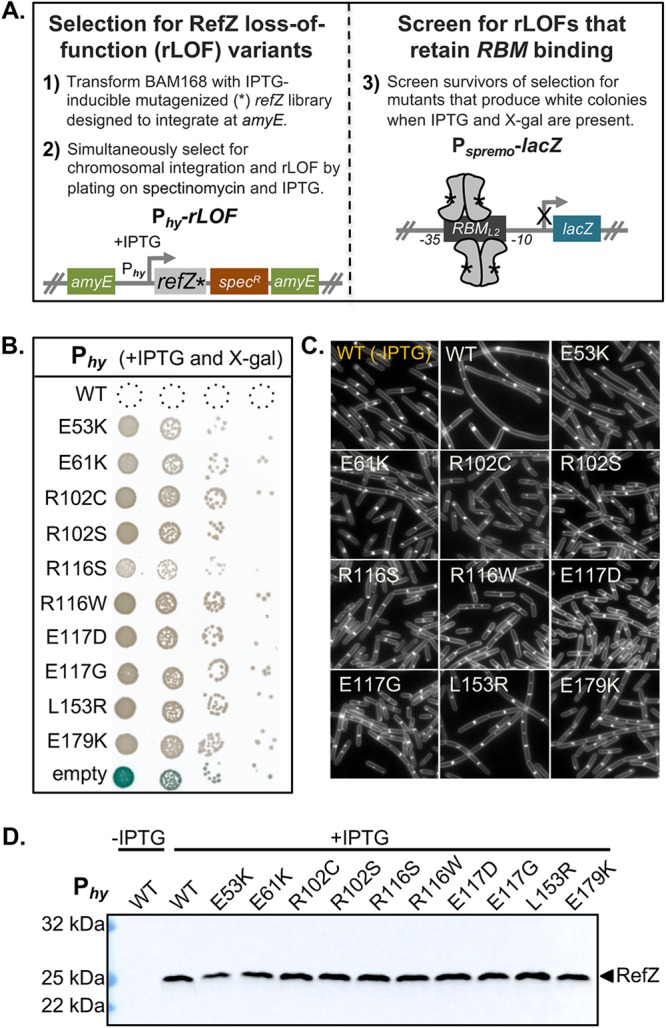
Isolation of rLOF variants. (A) Schematic of genetic selection (left) and screen (right) used to isolate rLOF variants that retain *RBM*-binding activity. The open reading frame of *refZ* was mutagenized by error-prone PCR (refZ*), placed under an IPTG-inducible promoter (P*_hy_*), and introduced at the *amyE* loci of competent recipient cells (BAM168). Mutations that interfere with RefZ’s division inhibition function (P*_hy_-rLOF*) permit growth in the presence of IPTG. Survivors were screened for *RBM* binding (P*_spremo_-lacZ*) on plates containing X-Gal and IPTG. (B) Ten unique rLOF variants that did not kill following induction but retained *RBM*-binding function were identified in the selection screen. (C) The rLOF artificial expression constructs were introduced into a wild-type (B. subtilis 168) genetic background, and the extent of cell filamentation in CH medium following 90 min of induction with 1 mM IPTG was monitored using epifluorescence microscopy. Membranes were stained with TMA (white). The uninduced WT control is labeled in yellow. (D) Western blot analysis to monitor the production and stability of wild-type RefZ (WT) and the rLOF variants following 45 min of induction with 1 mM IPTG. RefZ was not produced at levels detectable above background with our antibody during vegetative growth (lane 1, uninduced) or sporulation (data not shown).

To test whether RefZ’s ability to inhibit cell division is required to support precise chromosome capture, we designed a two-stage genetic selection screen to isolate rLOF variants capable of binding to the *RBM*s but unable to disrupt cell division upon artificial expression (Fig. 1). Gibson assembly (47) was used to generate a library of linear artificial expression constructs comprised of an IPTG (isopropyl-β-d-thiogalactopyranoside)-inducible promoter (P*_hy_*), randomly mutagenized *refZ* sequences (refZ*), a selectable marker (Spec^r^), and regions of homology to direct double-crossover integration of the linear DNA at a nonessential locus (*amyE*) (Fig. 1A). To select for rLOF mutants, we took advantage of the fact that in a sensitized background (Δ*minD*), expression of wild-type *refZ* from an IPTG-inducible promoter prevents colony formation on solid medium, whereas expression of RefZ variants unable to inhibit cell division allows survival (43). In addition to *minD*, the native *refZ* gene was also deleted to ensure that the only RefZ expressed would be from the inducible promoter.

To eliminate variants unable to bind DNA, survivors of the selection were screened for *RBM*-binding activity using a RefZ-repressible *lacZ* transcriptional fusion (P*_spremo_-lacZ*) integrated at the nonessential *sacA* locus. P*_spremo_* harbors a single *RBM* (*RBM_L2_*) (42) inserted between the −35 and −10 elements of a constitutive promoter (Fig. 1A). In this background, rLOF variants that can bind the engineered *RBM* operator repress *lacZ* expression and produce white colonies on media containing X-Gal (5-bromo-4-chloro-3-indolyl-β-d-galactopyranoside). In contrast, rLOF variants unable to bind the *RBM* due to decreased affinity for the *RBM*, poor expression, truncation, or misfolding produce blue colonies, allowing them to be excluded from further investigation.

To facilitate selection and screening efficiency and avoid cloning steps, transformation conditions were optimized so that the mutant *refZ* artificial expression construct library could be directly introduced into the B. subtilis chromosome (see Materials and Methods). RefZ loss of function and double-crossover integration were selected for simultaneously by plating transformants on a medium containing both spectinomycin and IPTG.

Approximately 1,300 viable transformants were obtained, 37 of which were either white or pale blue on medium containing X-Gal and IPTG, consistent with rLOF repression of *lacZ* expression from the engineered *RBM* operator. Since resistance to RefZ can also be conferred by spontaneous suppressor mutations in *ftsZ* (43), the 37 artificial expression constructs were transformed into a clean selection screen background, and survival and *RBM* binding were reassessed. Four candidates failed to survive on IPTG plates, suggesting the presence of suppressor mutations in the original strains, while an additional eight turned blue or light blue on X-Gal indicator medium.

**FIG 2:**
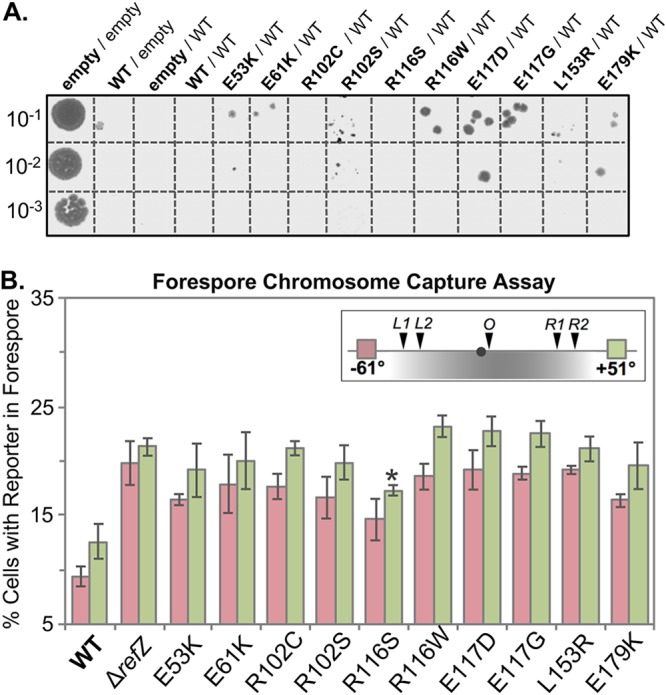
Functional characterization of rLOF variants. (A) *amyE*::*yhdG*::P*_hy_-refZ* (WT) was introduced into the chromosomes of recipient strains (boldface) harboring either inducible wild-type *refZ* (**WT**/WT), an rLOF mutant (**rLOF**/WT), or an empty vector (**empty**/WT). As a controls, an empty vector was introduced into the *yhdG* locus of the *amyE*::P*_hy_-empty* (**empty**/empty) and *amyE*::P*_hy_-refZ* (**WT**/empty) backgrounds. The resulting strains were grown in lysogeny broth at 30°C until mid-log phase. Cultures were normalized to the lowest OD_600_ reading in PBS (10^0^) and serially diluted to 10^−3^. Five microliters of the indicated dilution was spotted on LB plates supplemented with phleomycin and 1 mM IPTG, followed by overnight incubation at 37°C. (B) Quantitative single-cell analysis of chromosome capture is represented as the average percentage of cells that captured either the left-arm (−61°; pink) or right-arm (+51°; green) reporter in the forespore at the time of polar division. The inset shows the locations of the reporters relative to the *RBM*s, with the region of chromosome typically captured in the forespore shaded gray. The black circle represents *oriC* (0°). All strains encoding rLOF variants miscapture the left- and right-arm reporters at levels statistically indistinguishable from that for the Δ*refZ* mutant control (*P* > 0.05), with the exception of the R116S variant. The R116S right-arm reporter exhibited an intermediate capture defect that was statistically different from both Δ*refZ* (asterisk; *P* = 3.9 × 10^−3^) and the wild type (*P* = 2.3 × 10^−3^). The error bars represent standard deviations.

To identify rLOF mutations in the remaining 25 candidates, the P*_hy_-rLOF* region was amplified from the genomic DNA and sequenced (see Table S4 in the supplemental material). Six candidates had more than one single-nucleotide polymorphism (SNP) and were not characterized further. Of the 19 remaining candidates, substitutions were identified in nine different residues. The strains harboring L114 and L123 substitutions showed *lacZ* expression after extended incubation and were not included in further analysis. The remaining 10 rLOF candidates possessed substitutions corresponding to those shown in Fig. 1B. In contrast to wild-type RefZ, artificial expression of the rLOF variants did not result in cell filamentation (Fig. 1C), consistent with a loss of ability to affect FtsZ. The inability of rLOF variants to inhibit cell division was not anticipated to be attributable to protein misfolding or insufficient expression, as each variant was able to repress *lacZ* expression from the *RBM* operator in the primary screen (Fig. 1B). Consistent with this conclusion, Western blot analysis of the rLOF variants demonstrated that they were stably expressed and present at levels comparable to those of wild-type RefZ following artificial expression (Fig. 1D). When the variants were coexpressed alongside wild-type RefZ, killing was mostly restored (Fig. 2A), suggesting that the activity of the rLOF variants is largely recessive to wild-type RefZ. From these data, we conclude that the 10 rLOF variants are perturbed in their ability to affect FtsZ function, either directly or indirectly.

### rLOF mutants miscapture the forespore chromosome.

A Δ*refZ* mutant and a strain harboring point mutations in all five *oriC*-proximal *RBM*s (*RBM_5mu_*) both exhibited a 2-fold increase in the frequency of left- and right-arm reporter capture compared to the wild-type controls (42). We hypothesized that if RefZ's ability to perturb FtsZ assembly is required to mediate precise chromosome capture, then the rLOF mutants would phenocopy the Δ*refZ* mutant with regard to chromosome trapping. To test this hypothesis, chromosome organization was monitored in sporulating cells expressing the rLOF variants from the native locus (native promoter) using a fluorescence-based trapping assay (14, 42). For each strain, the native *refZ* gene was replaced with an rLOF mutant sequence in backgrounds harboring reporters for either left-arm (−61°) or right-arm (+51°) capture (Fig. 2B). All of the rLOF mutations resulted in significant increases in both left- and right-arm reporter capture compared to wild-type controls (*P* < 0.05) (Fig. 2B). Moreover, with the exception of right-arm capture in the R116S mutant, miscapture of both left- and right-arm reporters in the rLOF mutants was statistically indistinguishable from the Δ*refZ* controls (*P* > 0.05). The right-arm reporter in the R116S mutant exhibited an intermediate capture defect that was statistically different from both Δ*refZ* (*P* = 3.9 × 10^−3^) and the wild type (*P* = 2.3 × 10^−3^). The intermediate capture defect observed in the R116S mutant suggests this variant retains some functionality and is consistent with the reduced growth we observed on selection medium in the sensitized Δ*minD* background (Fig. 1B). These data demonstrate that the same residues required for RefZ’s ability to inhibit division upon artificial expression are also required for precise chromosome capture, and are consistent with a model in which *RBM*-bound RefZ modulates FtsZ activity to position the polar septum relative to the chromosome.

### Structural characterization of RefZ.

**FIG 3:**
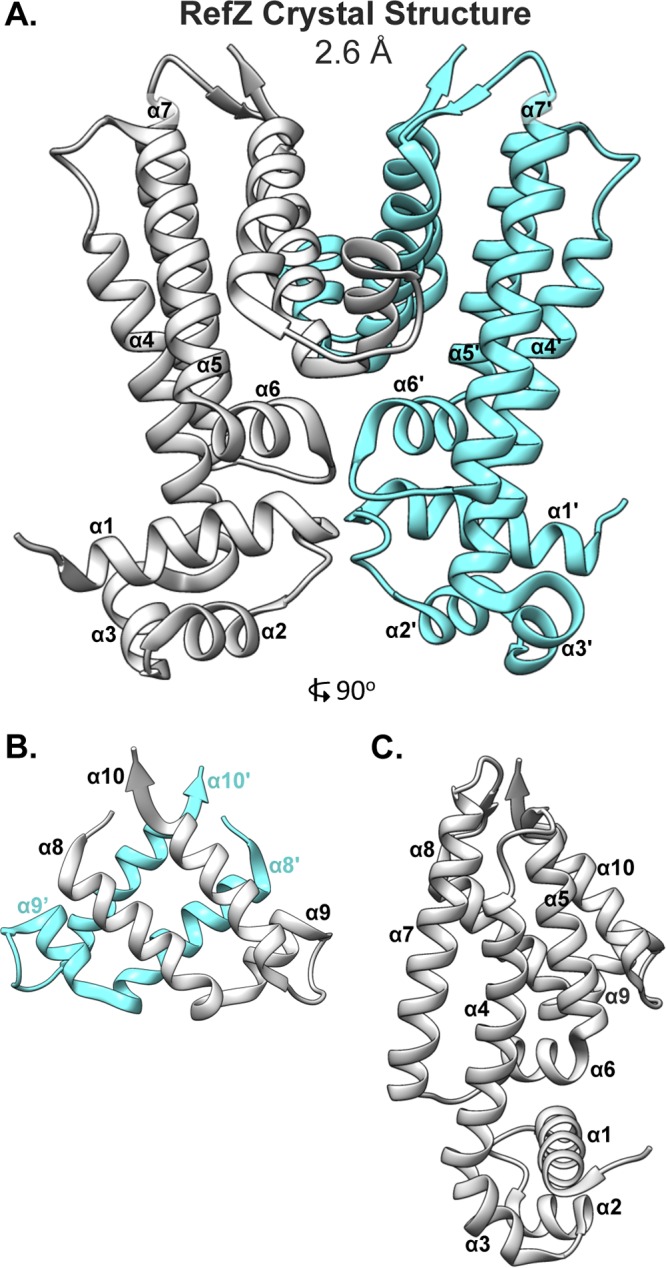
Crystal structure of the RefZ homodimer at 2.6-Å resolution. (A) Structure of the RefZ homodimer. The subunits are colored gray and cyan. (B) Helices α8 to α10 of RefZ’s regulatory region, with antiparallel helices α8, α10, α8′, and α10′ comprising the four-helix dimerization motif. (C) The RefZ monomer, rotated 90° relative to panel A.

Like the E. coli NO protein, SlmA, RefZ belongs to the TetR family of DNA-binding proteins (43). At the sequence level, RefZ and SlmA share no significant similarity. We reasoned that structural characterization of RefZ and mapping of the rLOF substitutions to the RefZ structure would not only provide insight into how RefZ functions but also allow comparison to what is known about SlmA’s mechanism of FtsZ inhibition. RefZ-His_6_ was purified and crystallized, and the structure was solved using single-wavelength anomalous dispersion (SAD) phasing at a resolution of 2.6 Å. RefZ crystallized as a homodimer (Fig. 3A) with one molecule in the asymmetric unit of a P4_1_2_1_2 crystal lattice. The model for residues 1 to 200 was built and refined with *R*_work_ equal to 22% and *R*_free_ equal to 25% (see Table S5 in the supplemental material). Each RefZ subunit is composed of 10 α-helices connected by loops and turns. Similar to other structurally characterized TetR family proteins (48), α1, α2, and α3 comprise the DNA-binding helix-turn-helix (HTH) domain and α4 to α10 comprise the regulatory domain (Fig. 3A). There are two major regions for dimerization contacts. Helices α7, α8, α9, and α10 form regulatory domain contacts with α7′, α8′, α9′, and α10′; α8, α10, α8′, and α10′ form a four-helix dimerization motif (Fig. 3B). A second interface is formed by α6 and α6′ at the junction between the regulatory and DNA-binding domains (Fig. 3A). Although the crystallization conditions included *RBM*-containing DNA, we observed no DNA in the crystal structure. In fact, the HTH DNA-binding domain is involved in extensive crystal-packing interactions, likely precluding DNA binding within the crystal lattice.

According to a structural similarity search using VAST (49), RefZ shares the highest homology with PfmR from Thermus thermophilus (Protein Data Bank identifier [PDB ID] 3VPR) (50), with a VAST similarity score of 15.4, closely followed by KstR2 of Mycobacterium tuberculosis (PDB ID 4W97) (51), with a score 15.2. The SlmA structure (PDB ID 4GCT) (52) was the 10th closest in similarity, with a score of 13.6. Superposition of SlmA and RefZ produced a root mean square deviation (RMSD) in Cα of 2.8.

RefZ’s HTH domain (residues 1 to 45) has the highest contiguous alignment similarity score with QacR from Staphylococcus aureus (PDB ID 1JT6) (53), with a VAST similarity score of 4.0 and an RMSD value of 0.7. Superimposition of the HTH domains demonstrates that the structures align closely (see Fig. S1A in the supplemental material). However, when the RefZ dimer is superimposed on DNA-bound QacR (PDB ID 1JT0), it is apparent that the RefZ dimer would need to undergo a conformational change for the α3 and α3' helices to be accommodated in adjacent DNA major grooves (see Fig. S1B and C).

DNA binding in TetR family proteins can be allosterically regulated by ligand binding in a pocket formed by α5, α6, and α7. For QacR, ligand binding results in a pendulum motion of α4 that repositions the HTH domains so that the distance between α3 and α3′ becomes incompatible with DNA binding (54). In the RefZ structure (unbound from DNA), there is no obvious ligand binding pocket in the α5-to-α7 regulatory region; therefore, its affinity with DNA is unlikely to be regulated in this manner. At the same time, we do not exclude the possibility.

### The regions of RefZ and SlmA important for inhibiting cell division are distinct.

**FIG 4:**
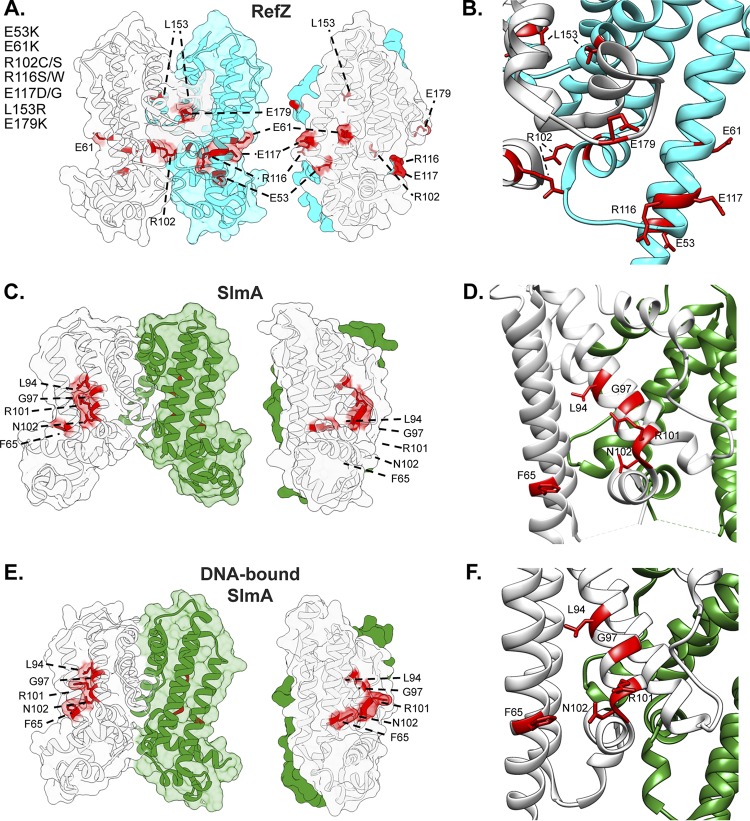
Positions of residues implicated in RefZ’s regulation of cell division. (A) Surface/cartoon representation of the RefZ homodimer highlighting residues with substitutions conferring loss of function (red; sticks). The subunits are colored white and cyan. (B) Ribbon model of the RefZ region showing residues conferring loss of function as sticks. (C and E) Surface/cartoon representations of the SlmA homodimer (unbound) (PDB ID 3NXC) (28) (C) and the SlmA homodimer bound to DNA and the CTD tail of FtsZ (PDB ID 5HBU) (26) (E), highlighting residues with substitutions conferring loss of function (red; sticks). The subunits are colored white and green. (D and F) Ribbon models corresponding to the SlmA homodimer (PDB ID 3NXC) (28) (D) and SlmA homodimer bound to DNA and the CTD tail of FtsZ (PDB ID 5HBU) (26) (F), showing residues conferring loss of function as sticks.

**FIG 5:**
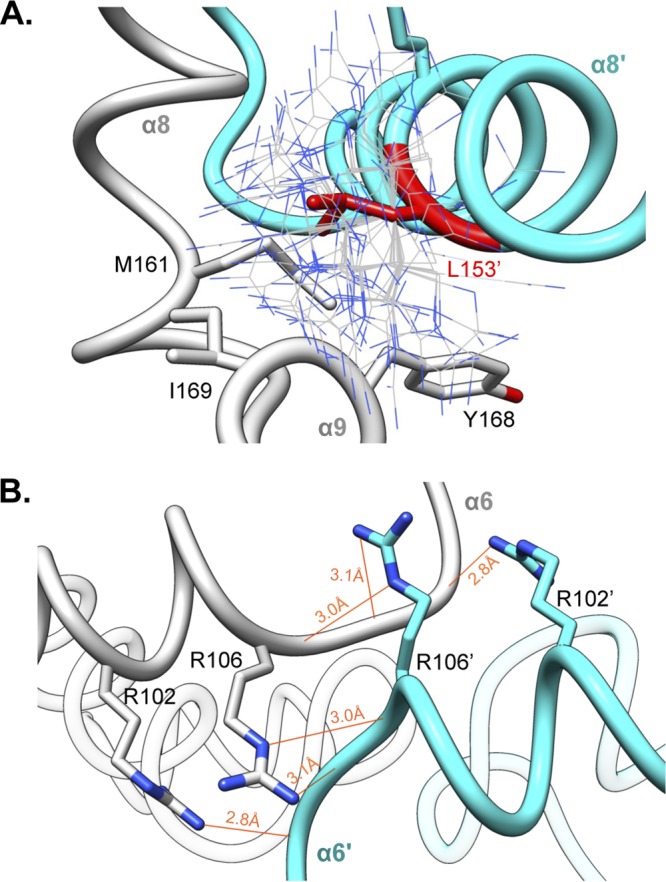
Dimer interface residues implicated in RefZ function. RefZ subunits are shown in light gray and cyan. (A) Hydrophobic dimerization interface near the L153 residue. The thin blue and gray sticks display possible positions of an R153 side chain based on a rotamer library. (B) Helices α6 and α6′ of RefZ, with residues implicated in loss of function shown as sticks. The hydrogen bonds formed across the dimer interface by R102 and R106 are displayed as red lines.

To analyze which regions of RefZ are important for its effect on cell division and to compare them to the location of the loss-of-function residues identified for SlmA, the residues with rLOF substitutions were mapped to the RefZ crystal structure (Fig. 4). Nine of the 10 rLOF substitutions (L153R is the exception) occur in charged residues that are surface exposed and map to the same surface of the RefZ homodimer (Fig. 4A and B). L153 maps to the dimerization interface (Fig. 5A) and participates in several hydrophobic interactions between subunits that are likely important for RefZ dimerization. Not only is R102 surface exposed, but also hydrogen bonds across the dimer interface to the backbone carbonyl of V108′ (NH_2_-O = 2.6 Å) (Fig. 5B).

To assess if similar regions of SlmA were implicated in FtsZ regulation, the structures of the RefZ and SlmA homodimers were compared (Fig. 4). In the DNA-bound structure, SlmA binds the C-terminal domain (CTD) tail of FtsZ along a hydrophobic groove located between α4 and α5 (26, 27). SlmA loss-of-function substitutions map to this region, clustering primarily along α4 (Fig. 4E and F) (26, 55). In contrast, the surface-exposed residues implicated in RefZ loss of function are positioned at or on either side of the RefZ dimerization interface, and all but L153 are positively or negatively charged (Fig. 4A). Moreover, the structures of the RefZ and the SlmA homodimers (unbound) adopt distinct conformations (Fig. 4A and C). From these data, we conclude that if RefZ regulates FtsZ through direct interaction, then the precise mechanism is likely to differ significantly from that of SlmA.

### Characterization of RefZ and rLOF variant DNA binding.

**FIG 6:**
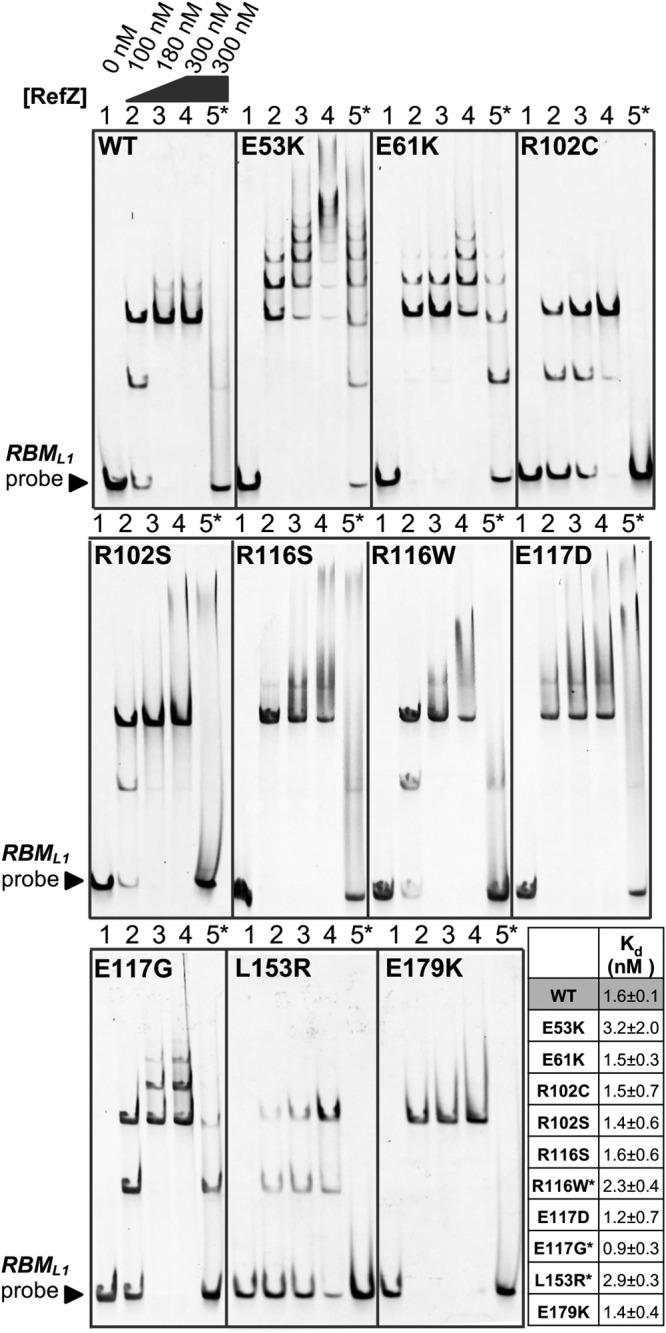
Interaction of the rLOF variants with DNA. Electrophoretic mobility shift assays were performed with 150-bp DNA probes (10 nM) centered on either the wild-type (lanes 1 to 4) or the mutant (lane 5*) *RBM_L1_* sequence. Probes were incubated with the indicated concentrations of purified RefZ-His_6_ (WT) or rLOF-His_6_ variants for 30 min. Reactions were run on a 5% TBE gel for 30 min at 150 V. The tabulated *K_d_* values of RefZ for an immobilized 41-bp *RBM*-containing DNA segment were determined using a biolayer interferometry assay. All the variants possessed *K_d_* values within 2-fold of the wild-type *K_d_*. The differences in *K_d_* between wild-type RefZ and R116W, E117G, and L153R are significant (indicated by asterisks) (*P* = 0.05, *P* = 0.025, and *P* = 0.003, respectively).

RefZ’s ability to inhibit cell division is dependent upon DNA binding (43). We predicted that the rLOF variants would be DNA-binding proficient because each was able to repress *lacZ* expression from an *RBM* operator in the *in vivo* screening assay (Fig. 1B); however, *RBM* binding in the assay was qualitative and was not designed to differentiate between specific and nonspecific DNA interactions. To directly examine the behavior of the variants with DNA, we overexpressed and purified each of the rLOF variants (see Fig. S2 in the supplemental material) and performed electrophoretic mobility shift assays (EMSAs) with wild-type and mutant *RBM* DNA probes as described previously (42). Incubation of wild-type RefZ with a 150-bp *RBM*-containing probe produced two major mobility shifts (Fig. 6) corresponding to RefZ binding to *RBM*-containing DNA in units of two and four. Consistent with previous observations (42), the upshifts were lost when RefZ was incubated with a mutant *RBM* probe (harboring seven point mutations in the central palindrome), indicating that DNA binding is specific to the *RBM* sequence (Fig. 6). Four of the rLOF variants (R116S, R116W, E117D, and E179K) produced specific upshifts similar to those with wild-type RefZ, suggesting that their loss-of-function phenotypes are not attributable to altered affinity or nonspecific DNA binding.

The remaining variants exhibited altered DNA interactions with respect to specificity and/or the mobility shift pattern. Two variants (E53K and E61K) exhibited a laddering pattern, possibly due to additional subunits of RefZ binding nonspecifically along the DNA (Fig. 6). These variants also shifted a mutant *RBM*, consistent with enhanced nonspecific binding. E53K and E61K may assume conformations more favorable for nonspecific DNA binding, since the substitutions are located on α4, a helix important for modulating DNA interaction in response to ligand binding in other TetR family members (56). Although the laddering behavior was most extensive with E53K and E61K mutants, wild-type RefZ was also observed to ladder slightly (Fig. 6). The laddering behavior is more apparent when the EMSA gels are run at a higher voltage (200 V versus 150 V) (see Fig. S3A in the supplemental material), likely because EMSAs are nonequilibrium assays and the faster run time reduces RefZ disassociation. E117G also produced laddering, albeit to a lesser extent than either E53K or E61K (Fig. 6). Each of the remaining variants, R102C, R102S, and L153R, possesses substitutions in residues that make dimerization contacts (Fig. 5). R102C, R102S, and L153R produced two major upshifts but were unable to ladder on DNA even under EMSA conditions under which wild-type RefZ displayed some laddering (see Fig. S3B).

To establish whether quantitative differences in DNA binding might account for the loss-of-function phenotypes, we determined the dissociation constants (*K_d_*) of wild-type RefZ and each of the rLOF mutants for a 41-bp segment of *RBM*-containing DNA using biolayer interferometry. The *RBM*-containing DNA, which was 5′ biotinylated, was immobilized on a streptavidin sensor. The association and dissociation of wild-type RefZ (see Fig. S3C) and the rLOF variants were then assessed by monitoring the change in thickness of the biolayer. All of the rLOF variants displayed *K_d_* values within 2-fold of the wild type (Fig. 6). The decreased *K_d_* for the L153R mutant was most significant (*P* < 0.01), consistent with the reduced apparent affinity for DNA observed by EMSA (Fig. 6). These results suggest that the *in vivo* chromosome capture defect observed in strains harboring rLOF mutations (Fig. 2B), with the possible exception of L153R, is unlikely to be attributable to markedly reduced affinity for DNA.

### RefZ oligomerization state by size exclusion chromatography.

**FIG 7:**
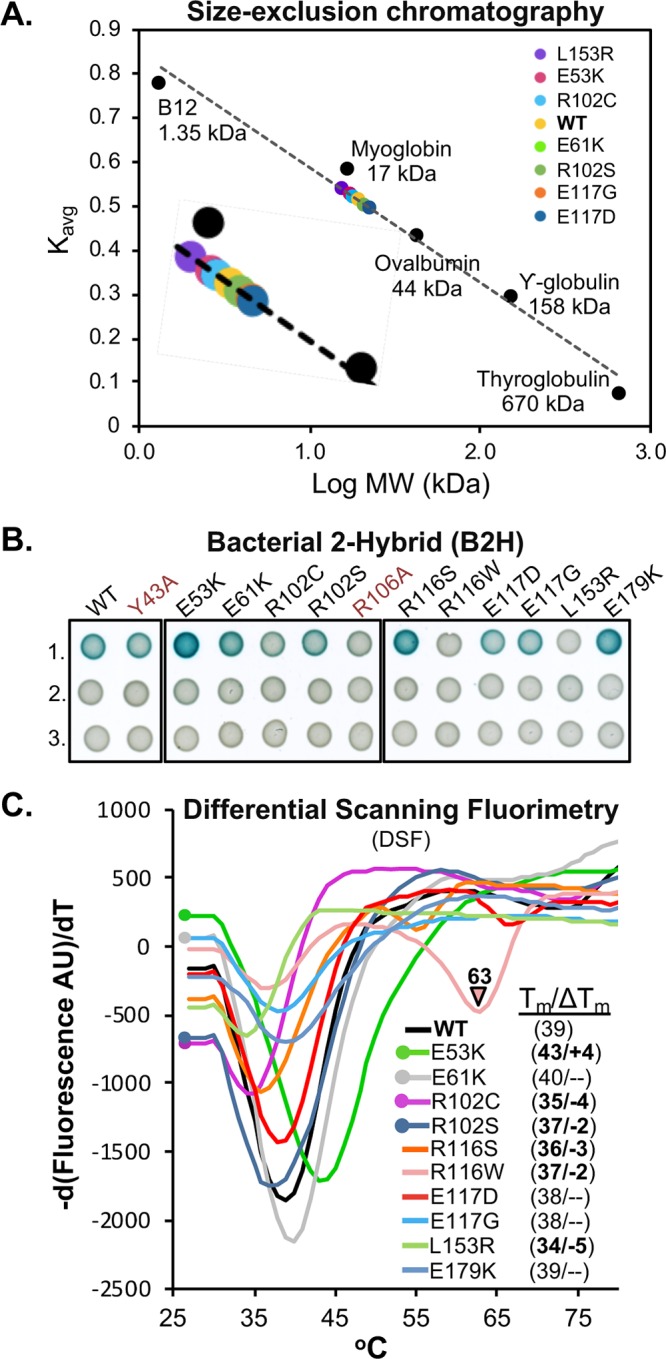
Oligomeric states and thermostability of wild-type RefZ and rLOF variants. (A) Size exclusion chromatography of wild-type RefZ-His_6_ and a subset of rLOF-His_6_ variants on a Superdex 200 column. The *K*_avg_ values for the indicated standards were used to generate a standard curve and to estimate the apparent molecular weights of the experimental samples. The E61K and R102C variants share the same position on the curve, and only R102C (cyan) is visible. (B) Self-interaction of wild-type RefZ or rLOF variants in a B2H assay. The RefZ variants in red (Y43A and R106A) were generated by site-directed mutagenesis and do not bind *RBM*-containing DNA. Wild-type RefZ subunits or the subunits of the indicated variants were fused to T25 and T18 tags. Shown are pairwise interactions between wild-type RefZ subunits or the subunits of the indicated variants fused to T25 and T18 tags (row 1), T25-tagged subunits paired with an empty T18 vector (row 2), or T18-tagged subunits paired with an empty T25 vector (row 3). Color development after 41 h of growth at room temperature is shown. (C) DSF of wild-type RefZ-His_6_ and the rLOF-His_6_ variants. Protein stability is reported by fluorescence of Sypro orange as a function of increasing temperature. *T_m_* values were calculated by determining the temperature at which the first derivative, d(Fluorescence AU)/dT, was at a minimum. Δ*T_m_* (inset) is the difference in *T_m_* values between wild-type RefZ and each rLOF variant. A Δ*T_m_* value of 1.5°C or less was not considered to be significant and is shown as a dash.

Three of the rLOF substitutions (R102C, R102S, and L153R) map to residues implicated in RefZ dimerization based on structural analysis (Fig. 5), suggesting dimerization may be important for RefZ’s effect on cell division. Purified TetR proteins have been shown to exist as both monomers and dimers in solution and as pairs of dimers on DNA (28, 56–59). RefZ also binds DNA in units of two and four (42), but its oligomerization state in the absence of DNA is unknown. To determine the oligomerization states of purified RefZ and the rLOF variants, we performed size exclusion chromatography. Wild-type RefZ-His_6_ eluted from a Superdex 200 column primarily as a single peak corresponding to an apparent molecular weight of 21 kDa, close to the actual monomeric molecular weight of 25.4 kDa (Fig. 7A; see Fig. S4 in the supplemental material). A minor peak, corresponding to an aggregate or higher-order oligomer, was also observed (see Fig. S4). All of the rLOF variants tested displayed elution profiles comparable to that of the wild type (Fig. 7A). These data indicate that if RefZ forms dimers in the absence of DNA under the buffer conditions utilized, then they are not stable enough to be maintained during size exclusion chromatography.

### Bacterial two-hybrid analysis of RefZ self-interaction.

Size exclusion chromatography is known to dissociate weaker oligomers, including dimers of at least one TetR family protein (54). Therefore, to further investigate if any of the rLOF substitutions altered RefZ’s ability to form dimers, we performed bacterial 2-hybrid (B2H) analysis (60). In the B2H assay, wild-type RefZ displayed a self-interaction that was not observed in the negative controls (Fig. 7B). The self-interaction is unlikely to require *RBM* binding, as the B2H assay is performed in an E. coli strain that lacks native *RBM* motifs. Consistent with this observation, a DNA-binding-deficient variant, Y43A (43), displayed self-interaction similar to that of the wild type (Fig. 7B). The B2H assay is most likely reporting on dimerization, as RefZ forms a homodimer in the crystal structure (Fig. 3A). To explore this possibility further, we introduced a substitution at the dimerization interface predicted to disrupt hydrogen bonding between RefZ subunits. Substitution of an alanine at R106, an invariant residue in *Bacillus refZ* homologs that participates in two hydrogen bond contacts across the dimer interface (four bonds in total) (Fig. 5B), resulted in reduced self-interaction, as expected (Fig. 7B).

B2H analysis of the 10 rLOF variants revealed three classes of self-interaction phenotypes: loss of interaction, gain of interaction, and wild-type interaction (Fig. 7B; see Fig. S5 in the supplemental material). Three rLOF variants, L153R, R102C, and R116W, were classed as loss of interaction. Like R106, R102 and L153 are located on the dimer interface. R102 contributes a total of two hydrogen bonds to RefZ dimer formation (Fig. 5B). Substitution of a cysteine at R102 would therefore be expected to reduce dimerization, and this is consistent with the reduced self-interaction observed (Fig. 7B). The L153R substitution introduces a longer, positively charged side chain into a hydrophobic region of the RefZ dimer interface and thus is also predicted to reduce dimerization (Fig. 5A). No self-interaction was observed for the L153R variant, consistent with the structural prediction. These data suggest that the loss-of-function phenotypes of R102C and L153R may be related to a reduced ability to dimerize.

Three variants, E53K, R116S, and E179K, displayed enhanced self-interaction compared to the wild type (Fig. 7B). E53K is positioned on α4, the helix connecting the regulatory domain (α4 to α10) to the DNA-binding domain (α1 to α3). In TetR and QacR, conformational changes to the regulatory domain caused by ligand binding are transmitted through α4 to the HTH, leading to DNA release (56). Since the E53K mutant also shows higher affinity for nonspecific DNA (Fig. 6), we hypothesize that E53K facilitates a conformation that both dimerizes and binds DNA more readily. Given that the R116S and R116W variants display opposite phenotypes (enhanced and weakened self-interaction, respectively), R116 clearly has an important role in determining RefZ’s dimerization state. The E179K substitution is located just proximal to α8, a helix that participates in hydrophobic interactions between RefZ subunits (Fig. 3B). The E179K substitution may cause a change in RefZ’s overall conformation that enhances hydrophobic interactions between helices α8 and α8′ of the RefZ subunits.

Four variants, R102S, E61K, E117D, and E117G, exhibited self-interaction comparable to that of the wild type (Fig. 7B). Notably, even though the R102S and E117D substitutions support wild-type self-interaction (Fig. 7B) and *RBM* binding (Fig. 6), they are not functional *in vivo*. These results suggest that R102 and E117 are perturbed in functions not revealed by the *ex vivo* assays. At the same time, since 6 of the 10 rLOF variants display either reduced or increased self-interaction, these data suggest that the ability of RefZ to switch between monomer and dimer forms is likely important for the mechanism leading to FtsZ inhibition.

### Thermostability of RefZ and the rLOF variants.

To examine the effects of the rLOF substitutions on RefZ’s thermostability, we performed differential scanning fluorimetry (DSF). Wild-type RefZ displayed a single-transition melting curve (see Fig. S6, WT), with a melting temperature (*T_m_*) of 39°C (Fig. 7C). With the exception of R116W, all of the variants displayed single-transition melting curves (see Fig. S6 in the supplemental material). Most of the variants exhibited a lower *T_m_* than the wild type (L153R < R102C < R116S < R102S < WT). Notably, L153R and R102C were the most destabilized (−5°C and −4°C, respectively) and also showed the weakest self-interaction in the B2H assay (Fig. 7B). Conversely, E53K was more thermostable than the wild type and also displayed the most self-interaction by B2H assay (Fig. 7C). R116W also displayed reduced thermostability and self-interaction; however, unlike L153R and R102C, the R116W melting curve displayed two transitions, suggesting that the R116W variant assumes more than one conformation in solution. These results suggest that RefZ and the rLOF variants may assume multiple conformations in solution and that RefZ’s oligomerization state may be partly reflected in the thermostability measurements.

## DISCUSSION

RefZ is required for the timely redistribution of FtsZ from midcell to the pole (43). RefZ can also inhibit Z ring assembly and filament cells when it is artificially induced during vegetative growth, an activity that requires DNA binding (43). Under its native regulation, RefZ is expressed early in sporulation and requires the *RBM*s to facilitate precise capture of the chromosome in the forespore (42). Together, these results suggest that RefZ's effect on FtsZ, whether direct or indirect, is regulated by interactions with the nucleoid. Strikingly, the *RBM*s and their relative positions on the chromosome with respect to *oriC* are conserved across the entire *Bacillus* genus, indicating there is strong selective pressure to maintain the location of the *RBM*s. In B. subtilis, the *RBM*s are positioned in the cell near the site of polar septation. These observations, and the fact that RefZ, like SlmA (the NO protein of E. coli), belongs to the TetR family of DNA-binding proteins led us to hypothesize that RefZ binds to the *RBM*s to tune Z ring positioning relative to the chromosome during sporulation.

To evaluate whether RefZ's FtsZ-inhibitory activity was important for chromosome capture, we took advantage of RefZ's vegetative artificial expression phenotype (filamentation and cell killing in a sensitized background) to isolate 10 rLOF variants capable of binding DNA but unable to inhibit cell division. All 10 of the rLOF variants were unable to support correct chromosome capture (Fig. 2B), consistent with a model in which RefZ-*RBM* complexes act through FtsZ to facilitate precise septum placement with respect to the chromosome during polar division. This model is also supported by recent evidence showing that, on average, Δ*refZ* mutants position Z rings approximately 15% further away from the cell pole than does the wild type (61).

### RefZ and SlmA do not inhibit FtsZ through a common mechanism.

To better understand RefZ's mechanism of action at the molecular level, wild-type RefZ and the rLOF variants were overexpressed, purified, and analyzed using structural and biochemical approaches (summarized in Table 1). The RefZ crystal structure revealed that RefZ is capable of forming a homodimer (Fig. 3), similar to other TetR proteins, including SlmA. The relative locations and natures of the loss-of-function substitutions in RefZ and SlmA are different (Fig. 4), suggesting that if RefZ interacts with FtsZ directly, then RefZ’s mechanism of action is distinct from that of SlmA. At least some mechanistic differences would be expected, as the C-terminal tails of the FtsZ proteins from B. subtilis and E. coli are distinct. More specifically, while the portion of E. coli FtsZ observed to interact with SlmA in the cocrystal is relatively conserved (DIPAFLR in E. coli and DIPTFLR in B. subtilis), the remainder of the C termini differ significantly (KQAD in E. coli and NRNKRG in B. subtilis).

**TABLE 1:** Summary of rLOF phenotypes

rLOF variant	Relative strength^*a*^ of:				Δ*T_m_* (°C)^*b*^
	EMSA laddering	*RBM* specificity	*K_d_*	Self-interaction	
WT	++	+++	++	++	NS
E53K	++++	+	++	++++	+4
E61K	++++	+	++	++	NS
R102C	+	+++	++	+	−4
R102S	+	+++	++	++	−2
R116S	++	+++	++	+++	−3
R116W	++	+++	+	ND	−2
E117D	++	+++	++	++	NS
E117G	+++	++	+++	++	NS
L153R	ND	+++	+	ND	−5
E179K	++	+++	++	+++	NS

a Compared to wild type. ND, not detected.

b A Δ*T_m_* of ≤1.5°C compared to wild type was considered not significant (NS).

### The roles of self-interaction and RBM binding in RefZ function.

An important finding of this study is that both enhanced and reduced RefZ dimerization are correlated with loss-of-function phenotypes *in vivo*. B2H analysis indicated that the majority of rLOF variants (6/10) exhibited either stronger or weaker self-interaction (Fig. 7B), suggesting that RefZ's propensity to switch between monomer and dimer states is integral to affecting FtsZ function. Two rLOF variants (R102C and L153R) possess substitutions predicted to disrupt dimerization (Fig. 5), a result corroborated by B2H analysis (Fig. 7B). L153R also causes a 2-fold reduction in affinity for *RBM*-containing DNA, which could affect its ability to appropriately localize to *RBM*s *in vivo*.

Two rLOF variants (E53K and E61K) are located on α4. Based on the observation that E53K and E61K exhibit enhanced laddering and an increased apparent affinity for nonspecific DNA by EMSA (Fig. 6), we propose that these variants assume a conformation that is more favorable for nonspecific DNA binding than the conformation assumed by the wild type. In vivo, enhanced nonspecific binding would reduce the formation of RefZ-*RBM* complexes, which prior data suggest is the functional form of RefZ (42, 43).

The ability of RefZ to generate DNA laddering in EMSAs (Fig. 6; see Fig. S3 in the supplemental material) is presumably due to the association of additional RefZ subunits to adjacent DNA after the initial pair of dimers binds the *RBM* (42). Other TetR proteins, including SlmA, have also been observed to “spread” on DNA *in vitro* (52, 57, 62). In the case of SlmA, spreading on DNA is hypothesized to facilitate interaction with the exposed C-terminal tails of FtsZ to promote filament breakage (52). Although genetic and cell-biological data suggest RefZ and FtsZ interact (42, 43, 61), evidence for direct interaction between RefZ and FtsZ is lacking. We were unable to detect a positive interaction between FtsZ and RefZ *in vivo* by bacterial 2-hybrid analysis (see Fig. S7). Our attempts to test for RefZ-FtsZ interaction *in vitro* were impeded by RefZ’s limited solubility in FtsZ polymerization buffers. Therefore, the precise mechanism by which RefZ affects FtsZ remains to be determined.

One of the most interesting observations obtained from characterizing the rLOF variants is that the R116S and R116W substitutions on the first turn of α7 result in opposite self-interaction phenotypes (Fig. 7B). The two variants behave comparably with regard to affinity and specificity for the *RBM*-containing DNA (Fig. 6), suggesting the loss-of-function phenotypes are not attributable to differences in DNA interaction or protein misfolding. Instead, the results suggest that R116 is a key residue in determining the stability of the RefZ dimer. We hypothesize that R116 participates in intramolecular bonds with residues within a flexible loop region (between α6 and α7; residues 109 to 114) (Fig. 3A), possibly contributing to the formation of a more stable homodimer. R116 could participate in the formation of either ionic or hydrogen bonds with an invariant aspartate residue (D111) located in the flexible loop. Our ability to assess R116’s role in intramolecular bond formation is limited in the current crystal structure, as the electron density for the R116 side chain is not well defined. Moreover, the electron density for the main chain of the flexible loop is moderately disordered, showing peaks of positive F_o_-F_c_ electron density next to the I110 and D111 side chains.

R116 is also immediately adjacent to E117, another critical residue identified in this study. E117D is the only rLOF variant that has loss of function with regard to inhibiting cell division and capturing the forespore chromosome yet is not detectably altered in the other RefZ properties implicated in function (Table 1). If RefZ targets FtsZ directly, then these data point toward E117 as a likely candidate residue for mediating interaction. The E117D substitution is intriguing, because the glutamate-to-aspartate change is highly conservative; however, if the interaction is direct, the shorter side chain of the aspartate could compromise RefZ’s ability to target FtsZ.

### Working model for RefZ-mediated septum positioning.

Based on the data available, we propose a model in which RefZ mediates chromosome capture by fine-tuning the position of FtsZ assembly over the forespore-destined chromosome. In our model, RefZ is primed to inhibit FtsZ polymerization near the pole by binding specifically to the polarly localized *RBM*s. Based on structural studies of other TetR family proteins and the observation that RefZ binds to *RBM*s in units of two and four *in vitro* (42, 43), RefZ likely binds each *RBM* as a pair of dimers. We were not able to report RefZ copy numbers, as native RefZ levels were too close to the detection limit of our antibodies; however, our preliminary data suggest that RefZ is likely a relatively low-copy-number protein.

Current data suggest the activity of RefZ inhibits rather than promotes FtsZ assembly (42, 43, 61). This raises the question as to how an inhibitor of FtsZ could act near the pole to promote precise placement of a polar division apparatus. In our model, RefZ is a locally acting inhibitor of FtsZ, and its primary function is not to inhibit the formation of polar Z rings altogether but rather to tune the location of Z ring assembly away from the immediate vicinity of the *RBM*s. Based on comparative analysis of the rLOF mutants, both decreased and increased abilities to dimerize appear to be detrimental to the inhibitory function of RefZ. This implies that a dynamic process of monomer-dimer exchange, not maintaining a specific oligomeric state, is what is important for RefZ function. One possibility is that *RBM*-bound dimers disassociate from DNA as monomers after engaging with FtsZ.

We present no evidence that RefZ’s DNA association or monomer-dimer exchange is influenced by a ligand, and no obvious ligand binding pocket was observed in the regulatory domain of the solved crystal structure. At the same time, we do not exclude the possibility that RefZ activity could be regulated through interaction with FtsZ or ligand binding. Recently EthR, an important TetR family protein from M. tuberculosis that regulates drug resistance, was shown to bind the nucleotide cyclic di-GMP (63). Interestingly, EthR’s proposed nucleotide binding region (based on mutagenesis and docking studies) is at the dimer interface, outside the canonical ligand binding pocket (near R102 in RefZ) (63).

Another paradox raised is why a Δ*refZ* mutant exhibits a slight delay in shifting Z rings from midcell to the pole during sporulation (43). If RefZ acts as an inhibitor at the pole, then assembly of the polar Z ring would be expected to accelerate in a Δ*refZ* mutant. This seeming contradiction may be explained by considering RefZ's localization during sporulation. At early time points, just before polar division occurs, RefZ-green fluorescent protein (GFP) localizes as foci near the poles. These foci likely represent RefZ-*RBM* complexes, as they are lost in a RefZ mutant that cannot bind DNA (43). Around the time polar division initiates, the polar RefZ foci become less apparent and RefZ is observed to coalesce near midcell at or near the membrane (43). The redistribution of RefZ's inhibitory activity from the pole to midcell as sporulation progresses could facilitate disassembly of the midcell Z ring and its reassembly at the pole (36, 37). One hypothesis raised by these data is that RefZ may have a second role—to prevent additional midcell divisions as sporulation progresses—and current investigations are aimed at exploring this possibility.

## MATERIALS AND METHODS

### General methods.

Strains, plasmids, and oligonucleotides are listed in Tables S1, S2, and S3 in the supplemental material, respectively. All B. subtilis strains were derived from B. subtilis 168 or PY79. Strain and plasmid construction is detailed in the supplemental material. Transformations in B. subtilis were carried out using a standard protocol, as previously described (64), unless otherwise stated. For selection in B. subtilis, antibiotics were included at the following concentrations: 100 μg ml^−1^ spectinomycin, 7.5 μg ml^−1^ chloramphenicol, 10 μg ml^−1^ kanamycin, 10 μg ml^−1^ tetracycline, 0.8 μg ml^−1^ phleomycin, and 1 μg ml^−1^ erythromycin (erm) plus 25 μg ml^−1^ lincomycin (macrolides-lincosamides-streptogramin B [MLS]). For transformation and selection in E. coli, antibiotics were included at the following concentrations: 100 μg ml^−1^ ampicillin, 25 μg ml^−1^ kanamycin, and 25 μg ml^−1^ chloramphenicol (for protein overexpression). Cotransformations for B2H assays were selected for on LB plates supplemented with 50 μg ml^−1^ ampicillin, 25 μg ml^−1^ kanamycin, and 0.2% (vol/vol) glucose.

### Two-step genetic-selection screen to isolate rLOF mutants.

Comprehensive details on construction of the Gibson assemblies and strains discussed below are available in the supplemental material. The *refZ* gene was mutagenized by error-prone PCR, and the mutant fragment library was introduced into an IPTG-inducible artificial expression construct using Gibson assembly (47). Multiple assembly reaction mixtures were pooled on ice and directly transformed into supercompetent BAM168 cells (selection screen background). For transformations, competent-cell aliquots were thawed at room temperature, and 0.2 ml was incubated in a 13-mm glass test tube with 20-μl assembly reaction mixtures for 90 min in a roller drum at 37°C before selecting on LB plates supplemented with 100 μg ml^−1^ spectinomycin and 1 mM IPTG. After overnight growth at 37°C, the surviving transformants were patched on LB plates supplemented with 1% (wt/vol) starch to screen for integration at *amyE* and on LB plates supplemented with the following antibiotics to assess the presence of the expected parental background resistances: 7.5 μg ml^−1^ chloramphenicol, 10 μg ml^−1^ kanamycin, 10 μg ml^−1^ tetracycline, and 1 μg ml^−1^ erythromycin plus 25 μg ml^−1^ lincomycin (MLS). Transformants were also patched on LB plates supplemented with 100 μg ml^−1^ spectinomycin, 1 mM IPTG, and 40 μg ml^−1^ X-Gal to screen for *lacZ* expression from the P*_spremo_* promoter. Replica plates were grown overnight at 37°C. Surviving rLOF mutants that did not turn blue on the patch plates were cultured from replica plates in liquid LB and stored at −80°C. Genomic DNA prepared from these strains was PCR amplified with OJH001 and OJH002 (see Table S3) to test for the presence of the expected integration product. PCR products of the expected size were sequenced to identify mutations.

### Generation of supercompetent cells.

Supercompetency was achieved using a 2-fold approach to maximize transformation efficiency. First, BAM168 cells (selection screen background) harbor a xylose-inducible copy of *comK* at the nonessential *lacA* locus (65). The presence of 1% (wt/vol) xylose in standard transformation cultures improved efficiency ∼2.5-fold compared to cultures grown without xylose. Second, competent cells were prepared by modifying an established (64) two-step B. subtilis competent-cell protocol as described below. The modifications improved transformation efficiency an additional 7-fold over xylose induction alone. A single colony of freshly streaked recipient cells (BAM168) was used to inoculate a 250-ml baffled flask containing 25 ml of 1× MC medium (10.7 g liter^−1^ K_2_HPO_4_, 5.2 g liter^−1^ KH_2_PO_4_, 20 g liter^−1^ glucose, 0.88 g liter^−1^ trisodium citrate dihydrate, 0.022 g liter^−1^ ferric ammonium citrate, 1 g liter^−1^ casein hydrolysate [Neogen], 2.2 g liter^−1^ potassium glutamate monohydrate, 3 mM MgSO_4_, and 0.02 g liter^−1^ l-tryptophan) (64). The culture was grown overnight (20 to 22 h) in a 37°C shaking water bath set at 250 rpm. The overnight culture (optical density at 600 nm [OD_600_], 1.5 to 2.5) was diluted to an OD_600_ of 0.1 in a 250-ml baffled flask containing 40 ml of 1× MC medium supplemented with 1% (wt/vol) xylose. The culture was incubated at 37°C in a shaking water bath set at 200 rpm. After 5 to 6 h of growth, the OD_600_ was monitored every 30 min until readings remained unchanged between two time points, at which point the culture was diluted 1:10 with prewarmed 1× MC medium supplemented with 1% (wt/vol) xylose to a final volume of 250 ml in a 2-liter flask. After 90 min of growth at 37°C and 280 rpm, cells were harvested at room temperature at 1,260 × *g* for 10 min in six 50-ml conical tubes. Twenty milliliters of the culture supernatant was retained and mixed with 5 ml 50% (vol/vol) glycerol. The diluted supernatant was used to gently resuspend the pellets, and the cell suspensions were immediately frozen at −80°C in aliquots.

### Blue-white screen to assess *RBM* binding by rLOF mutants.

Artificial expression constructs harboring either wild-type *refZ* (BAM374), rLOF mutants (BAM400, -403, -407, -409, -411, -440, -443, -444, -449, and -462), or an empty P*_hy_* vector (BAM390) in clean selection screen backgrounds (see the supplemental material) were streaked from frozen glycerol stocks on LB plates supplemented with 100 μg ml^−1^ spectinomycin and 0.2% (vol/vol) glucose and grown overnight at 37°C. Single colonies were used to inoculate 3 ml of lysogeny broth (LB-Lennox), and the cultures were grown in a roller drum at 30°C until early to mid-log phase (3 to 5 h). The cultures were normalized to the lowest OD_600_ with phosphate-buffered saline (PBS) (10^0^) and serially diluted (10^−1^, 10^−2^, and 10^−3^). Five microliters of each dilution was spotted on LB plates supplemented with 100 μg ml^−1^ spectinomycin, 1 mM IPTG, and 40 μg ml^−1^ X-Gal, followed by overnight incubation at 37°C to visually screen for *lacZ* expression from the P*_spremo_* promoter. The plates were scanned with a ScanJet G4050 flatbed scanner (Hewlett Packard) using VueScan software and medium format mode. Images were processed using Adobe Photoshop (version 12.0).

### rLOF dominance growth assay.

A wild-type copy of *refZ* under an IPTG-inducible P*_hy_* promoter was introduced at the ectopic *yhdG* locus of each of the IPTG-inducible variant strains listed in “Blue-white screen to assess *RBM* binding by rLOF mutants” above as described in the supplemental material. As controls, an empty P*_hy_* vector was introduced at the *yhdG* locus of the wild-type *amyE*::P*_hy_-refZ* (BAM374) and the *amyE*::P*_hy_*-empty vector (BAM390) strains. The resulting strains, BAM1662 to -1676, were streaked from frozen glycerol stocks on LB plates supplemented with 0.8 μg ml^−1^ phleomycin and 0.2% (vol/vol) glucose and grown overnight at 30°C. Single colonies were used to inoculate 3 ml of lysogeny broth (LB-Lennox), and the cultures were grown in a roller drum at 30°C until early to mid-log phase (3 to 5 h). Cultures were normalized to the lowest OD_600_ with PBS (10^0^) and serially diluted (10^−1^, 10^−2^, and 10^−3^). Five microliters of each dilution was spotted on LB plates supplemented with 0.8 μg ml^−1^ phleomycin and 1 mM IPTG, followed by overnight incubation at 37°C to visually screen for wild-type RefZ toxicity in the presence or absence of the rLOF variants. The plates were scanned with a ScanJet G4050 flatbed scanner (Hewlett Packard) using VueScan software and medium format mode. Images were processed using Adobe Photoshop (version 12.0).

### Artificial expression of wild-type *refZ* and rLOF variants.

Artificial expression constructs harboring either wild-type *refZ* (BJH228) or the rLOF mutants (BAM428, -431, -434, -436, -450, -451, -454, -455, -457, and -490) in a wild-type background (see the supplemental material) were streaked from frozen glycerol stocks on 100 μg ml^−1^ spectinomycin plates and grown overnight at 37°C. CH cultures (25 ml) were prepared as described in “Fluorescence microscopy” below. Expression was induced with 1 mM IPTG following 1.5 to 2 h of growth at 37°C (OD_600_, approximately 0.10). For the uninduced controls shown in Fig. 1C and D, an independent culture of the control strain, BJH228 (P*_hy_-refZ*), was grown in parallel but was not induced. Growth was resumed at 37°C with shaking for 45 min (see “Western blotting” below) or 90 min (see “Fluorescence microscopy” below) before 1-ml samples were harvested.

### Fluorescence microscopy.

For microscopy experiments, isolated colonies were used to inoculate 5 ml CH, and cultures were grown overnight at room temperature in a roller drum. Cultures below an OD_600_ of 0.7 were used to inoculate 25 ml CH medium in 250-ml baffled flasks to a calculated OD_600_ of 0.006 (for artificial expression) or 0.018 (for chromosome capture assays), and the cultures were grown for the indicated times at 37°C in a shaking water bath set at 280 rpm. Samples were collected at 6,010 × *g* for 1 min in a tabletop microcentrifuge. Following aspiration of the supernatants, the pellets were resuspended in 3 to 5 μl of 1× PBS containing 0.02 mM 1-(4-(trimethylamino)phenyl)-6-phenylhexa-1,3,5-triene (TMA-DPH) (Life Technologies), and cells were mounted on glass slides with polylysine-treated coverslips. Images were captured and analyzed with NIS Elements Advanced Research (version 4.10) software, using 600-ms (cyan fluorescent protein [CFP]), 900-ms (yellow fluorescent protein [YFP]), or 1-s (TMA) exposure times on a Nikon Ti-E microscope equipped with a CFI Plan Apo lambda DM 100× objective; a Prior Scientific Lumen 200 illumination system; C-FL UV-2E/C DAPI (4′,6-diamidino-2-phenylindole), C-FL YFP HC HISN zero shift, and C-FL cyan GFP filter cubes; and a CoolSnap HQ2 monochrome camera.

### Western blotting.

Samples were harvested at 21,130 × *g* for 1 min in a tabletop centrifuge. The pellets were washed with 50 μl of 1× PBS, and the remaining supernatant was carefully removed using a P20 pipette. The pellets were frozen at −80°C until they were processed. The frozen pellets were thawed on ice before resuspension in 25 μl of lysis buffer (20 mM Tris [pH 7.5], 10 mM EDTA, 1 mg ml^−1^ lysozyme, 10 μg ml^−1^ DNase I, 100 μg ml^−1^ RNase A, and 1 mM phenylmethylsulfonyl fluoride). Samples were normalized by OD_600_ values obtained at the time of harvest by diluting the resuspensions in additional lysis buffer before incubating them at 37°C for 15 min. Samples were diluted 1:1 with 2× sample buffer (250 mM Tris [pH 6.8], 10 mM EDTA, 4% [vol/vol] SDS, 20% [vol/vol] glycerol, and 10% [vol/vol] 2-mercaptoethanol) and boiled for 10 min. Five microliters of each lysate was loaded on a 4% to 20% gradient polyacrylamide gel (Lonza), and proteins were separated by electrophoresis prior to transfer to a nitrocellulose membrane (Pall) (1 h at 60 V). The membranes were blocked for 1 h at room temperature in 5% (wt/vol) nonfat milk in PBS (pH 7.4) with 0.05% (vol/vol) Tween 20. The membranes were incubated overnight at 4°C with polyclonal rabbit anti-RefZ antibody (Covance) diluted 1:1,000 in 5% (wt/vol) nonfat milk in PBS (pH 7.4) with 0.05% (vol/vol) Tween 20. The membranes were washed prior to a 1-h room temperature incubation with horseradish peroxidase-conjugated goat anti-rabbit immunoglobulin G secondary antibody (Bio-Rad) diluted 1:10,000 in 5% (wt/vol) nonfat milk in PBS (pH 7.4) with 0.05% (vol/vol) Tween 20. The washed membranes were incubated with SuperSignal West Femto maximum-sensitivity substrate (Thermo Scientific) according to the manufacturer’s instructions. Chemiluminescence was detected and imaged using an Amersham Imager 600 (GE Healthcare). The images were processed using ImageJ64 (66).

### Chromosome capture assay with the rLOF mutants.

Strains used in the chromosome capture assay (Fig. 2B) harboring the left-arm (−61°; P*_spoIIQ_-cfp*) or right-arm (+51°; P*_spoIIQ_-cfp*) reporter in the wild type, the *refZ* mutant, or the rLOF mutant trapping background (see Table S1) were streaked from frozen stocks on LB agar plates and grown overnight at 37°C. Chromosome capture assays were carried out as previously described (14, 42). CH cultures (25 ml) were prepared as described in “Fluorescence Microscopy” above and grown for 2.5 to 3 h (OD_600_, 0.6 to 0.8) before sporulation was induced by resuspension according to the Sterlini-Mandelstam method (64). Growth was resumed at 37°C in a shaking water bath for 2.5 h prior to TMA-DPH, YFP, and CFP image acquisition (see “Fluorescence microscopy” above).

Each strain harbored a σ^F^-dependent *oriC*-proximal reporter (−7°; P*_spoIIQ_-yfp*) that was captured in the forespore in 99.5% of sporulating cells. Cells expressing YFP served as the baseline for total sporulating cells counted in the field. To visualize cells in a given field that expressed the left- or right-arm reporters in the forespore, captured YFP and CFP images were individually merged with the TMA (membrane) image. The total number of forespores with YFP signal (total YFP) or CFP signal (total CFP) were manually marked and counted as described previously (42).

For quantitation and statistical analysis, a minimum of 1,500 cells per strain were counted from three independent biological and experimental replicates, with the exception of the wild type (left and right arms; *n* = 7) and the E53K strain (right arm; *n* = 4). The average proportion of cells expressing both reporters for each strain is given in Fig. 2, with error bars representing 1 standard deviation above and below the average. *P* values were determined using two-tailed, unpaired Student *t* tests.

### Protein purification.

E. coli BL21(DE3) pLysS competent cells were transformed with either pLM025a (RefZ-His_6_) or pEB013-pEB022 (rLOF-His_6_) and grown overnight at 37°C on LB plates supplemented with 25 μg ml^−1^ kanamycin, 25 μg ml^−1^ chloramphenicol, and 0.1% (vol/vol) glucose. Transformants were scraped from the plates and resuspended in 2 ml of cinnabar high-yield protein expression medium (Teknova) containing 25 μg ml^−1^ kanamycin, 25 μg ml^−1^ chloramphenicol, and 0.1% (vol/vol) glucose. Cells were vortexed, and the suspension was used to inoculate 100 ml of the same medium to a final OD_600_ of 0.1 and then divided equally into four 250-ml baffled flasks (25 ml/each). The cultures were grown at 37°C in a shaking water bath at 280 rpm for 6 to 7 h until the culture density reached an OD_600_ of 5.0. Protein expression was induced with 1 mM IPTG, and growth was resumed for an additional 3 h before the cultures were harvested by centrifugation at 9,639 × *g* for 5 min at 4°C. The pellets were stored at −80°C until they were processed. Four pellets (25 ml of culture each) were resuspended in 40 ml of lysis buffer (50 mM Tris-HCl [pH 9.0], 300 mM KCl, 10% [vol/vol] glycerol, and 10 mM imidazole); 1 μl protease inhibitor (Sigma-Aldrich; catalog no. P8465; 215 mg powder dissolved in 1 ml of dimethyl sulfoxide [DMSO] and 4 ml double-distilled H_2_O [ddH_2_O]) was added per 35 OD_600_ units. DNase I was added to a final concentration of 1 μg ml^−1^ of cell suspension. The suspensions were passed through a Microfluidizer LM20-30 (Microfluidics) five times at 10,000 lb/in^2^. Cell debris was cleared by centrifugation at 22,662 × *g* for 30 min at 4°C. The supernatants were passed over a 1-ml bed volume of nickel-nitrilotriacetic acid (NTA)–agarose beads (Qiagen; catalog no. 30210) preequilibrated with lysis buffer. The bound protein was washed with 10 ml of wash buffer (50 mM Tris-HCl [pH 9.0], 300 mM KCl, 10% [vol/vol] glycerol, and 20 mM imidazole). Protein was eluted with 7 ml of elution buffer (50 mM Tris-HCl [pH 9.0], 300 mM KCl, 10% [vol/vol] glycerol, and 250 mM imidazole) and collected as ∼250-μl fractions; 2 μl was removed from each fraction for SDS-PAGE analysis, and the elution mixtures were immediately stored at −80°C. Peak elution fractions were thawed and pooled before dialyzing at 4°C with stirring into either elution buffer (50 mM Tris-HCl [pH 9.0], 300 mM KCl, 10% [vol/vol] glycerol, and 250 mM imidazole) or ddH_2_O using Slide-A-Lyzer 7.0-kDa molecular weight cutoff (MWCO) dialysis cassettes (Thermo Fisher Scientific). Final protein concentrations were quantified using Bradford reagent (Bio-Rad) and a bovine serum albumin (BSA) standard.

### Protein crystallization, data collection, and data analysis.

RefZ-His_6_ was overexpressed and purified as described above. Before dialysis, the RefZ concentration was determined, and double-stranded DNA (dsDNA) (generated by annealing OEB025/OEB026 [see Table S3]) was added to a 4:1 molar ratio of RefZ-*RBM_L2-24bp_*. The protein was dialyzed into 50 mM Tris-HCl (pH 8.5) and 300 mM KCl. After dialysis, RefZ was concentrated in a 10-kDa Vivaspin Turbo MWCO filter (Sartorius) to ∼5 mg ml^−1^, and 0.5 to 1.0 μl of the concentrated protein was used to set crystallization plates. RefZ crystals formed within 48 h by hanging-drop vapor diffusion at 16°C after mixing the protein in a 1:1 (vol/vol) ratio with 10% ethanol, 0.1 M imidazole (pH 8.0), and 0.2 M MgCl_2_. The crystals were cryoprotected in 20% (vol/vol) glycerol in mother liquor before flash freezing in liquid nitrogen. For anomalous signal, RefZ crystals were soaked with 1 mM lead acetate for 5 h, and the data were collected at the Argonne National Laboratory APS synchrotron, beamlines 23-ID, at 0.9496 Å. Diffraction data were indexed, integrated, and scaled in HKL2000 (67), and the single heavy-atom site was identified by phasing using SAD in the SHELX program (68). The resultant phases were extended to a native crystal data set collected at the same beamline at 0.98 Å. The native set was indexed, integrated, and scaled using PROTEUM3 software (version 2016.2; Bruker AXS Inc.). The native crystal data were truncated in Ctruncate (69) from the CCP4 suite (70) and subjected to iterative building and phase improvement with PHENIX (71). The partial model produced by PHENIX was rebuilt in BUCCANEER (72) relying on improved phases. BUCCANEER was able to build the whole model in one continuous chain, docked in sequence and covering residues 1 to 200. The model was improved through iterative runs of inspection and manual modification in COOT (73) and refinement in PHENIX (71) with simulated annealing on initial runs. The data collection and refinement statistics can be found in Table S5.

### Annealing of oligonucleotides to generate dsDNA.

Oligonucleotides were resuspended in annealing buffer (10 mM Tris-HCl [pH 7.5], 50 mM NaCl, and 1 mM EDTA) to a concentration of 1 mM. Equal volumes were mixed and annealed in a thermocycler by heating to 95°C for 2 min followed by ramp cooling for 45 min to 25°C. The annealing buffer was removed by dialysis into ddH_2_O with Slide-A-Lyzer 7.0-kDa MWCO dialysis cassettes (Thermo Scientific).

### Electrophoretic gel mobility shift assays.

DNA fragments centered on either the native (using B. subtilis 168 as a template) or the mutant (using BJH205 as the template) *RBM_L1_* sequence (42) were generated by PCR using primer pair OEB009 and OEB010. Purified RefZ-His_6_ or rLOF-His_6_ protein (final concentrations are indicated in Fig. 6) were incubated with 10 nM *RBM_L1_* or *RBM_L1mu_* DNA probes in binding buffer (150 mM KCl and 10 mM Tris-HCl [pH 8.0]) for 30 min. After 30 min of incubation, 10× loading buffer (50 mM EDTA [pH 8.0], 1 mM Tris-HCl [pH 8.0], and 45% [vol/vol] glycerol) was added to a final concentration of 1×, and binding reactions were resolved at room temperature on a 5% Tris-borate-EDTA (TBE) polyacrylamide gel run for 45 min at 150 V (Fig. 6) or a 7.5% TBE polyacrylamide gel for 17 min at 200 V (see Fig. S3A and B). After electrophoresis, the gels were incubated with agitation in 1× SYBR green EMSA gel stain (Life Technologies; diluted from 10,000× stock in TBE buffer) for 5 min and then rinsed with distilled H_2_O (dH_2_O). The stained DNA was imaged with a Typhoon FLA 9500 scanner (General Electric) using the setting for fluorescence and an LPB (510LP) filter for SYBR green. The data presented in Fig. 6 are representative of a minimum of three independent experimental replicates for the wild type and each variant.

### Biolayer interferometry assay.

The Octet system (Pall Forte Bio) was used to monitor the kinetic interactions between wild-type RefZ or the rLOF variants and *RBM*-containing DNA. Streptavidin biosensors (part no. 18-5019) were purchased from Pall Forte Bio. A 41-bp *RBM*-containing (*RBM_L1_*) segment of dsDNA was generated by annealing 5′-biotinylated OEB091 with OEB092 as described in “Annealing of oligonucleotides to generate dsDNA” above, except that the annealing buffer was not removed by dialysis. All subsequent assays were performed in DNA-binding buffer (150 mM KCl and 10 mM Tris-HCl [pH 8.0]). Sensors were preequilibrated for 10 min at room temperature in DNA-binding buffer to establish a baseline reading. The sensors were then dipped into a well containing 50 nM *RBM_L1_* dsDNA and incubated for 2 min with shaking at 1,000 rpm to immobilize the DNA on the biosensor. The sensor was washed for 30 s to establish a new baseline before transfer to a solution containing 800 nM wild-type RefZ or rLOF variants. Following a 3-min monitored association, the complex was placed into fresh buffer, and dissociation was monitored continuously for 15 min. The *K_d_* was calculated using the global fit in Pall Forte Bio’s analysis software. Three experimental replicates of each assay were performed, except for variant R102C (*n* = 4). The mean values and standard deviations are given in Fig. 6. *P* values were determined using two-tailed, unpaired Student *t* tests.

### Size exclusion chromatography.

A Superdex 200 PC 3.2/30 3.2- by 300-mm column was equilibrated with 50 mM Tris-HCl (pH 9.0), 300 mM KCl, and 10% (vol/vol) glycerol. Wild-type RefZ and rLOF proteins from frozen stocks (ddH_2_O) were diluted to a final concentration of 1 mg ml^−1^ in 200 μl of buffer (50 mM Tris-HCl [pH 9.0], 300 mM KCl, 10% [vol/vol] glycerol). Samples were prespun at 21,130 × *g* for 10 min at 4°C in a tabletop centrifuge prior to injection. The absorbance at 280 nm was continuously measured, and the peak maximum (*V_e_*) was taken from the resulting elution profile and used to calculate the *K*_avg_ using the formula (*V_e_* – *V_o_*)/(*V_t_* − *V_o_*). The void volume (*V_o_*) was experimentally determined to be 7 ml. The total volume (*V_t_*) of the column was 24 ml. The apparent molecular mass was estimated using a curve generated from an identical run with a molecular mass standard (Bio-Rad Gel filtration chromatography standard; catalog no. 151-1901).

### Bacterial 2-hybrid analysis.

Assays were carried out essentially as previously described (42, 60). Plasmids harboring wild-type *refZ* and the rLOF sequences fused with C-terminal T18 and T25 tags (see the supplemental material for plasmid construction) were cotransformed into competent *E. coli* DHP1 (*cya* mutant) cells with selection on LB plates supplemented with 50 μg ml^−1^ ampicillin, 25 μg ml^−1^ kanamycin, and 0.2% (vol/vol) glucose. Cotransformed *E. coli* strains were streaked from frozen stocks, and single colonies were cultured in 4 ml of LB supplemented with 50 μg ml^−1^ ampicillin, 25 μg ml^−1^ kanamycin, and 0.1% (vol/vol) glucose in a 37°C roller drum to mid-logarithmic growth phase. Culture samples were normalized to the lowest-OD culture with fresh LB supplemented with 50 μg ml^−1^ ampicillin and 25 μg ml^−1^ kanamycin, and 5 μl was spotted on M9-glucose minimal plates supplemented with 50 μg ml^−1^ ampicillin, 25 μg ml^−1^ kanamycin, 250 μM IPTG, and 40 μg ml^−1^ X-Gal. Pairwise interactions between the T18 and T25 fusions were assessed by monitoring the development of blue color (corresponding to *lacZ* expression) following 40 to 50 h of growth at room temperature. Figure 7B is representative of three independent biological and experimental replicates (see Fig. S5). Interaction between FtsZ and RefZ (see Fig. S7 in the supplemental material) was assayed for as described above. Spot plates were grown at room temperature for 46 h prior to imaging.

### Differential scanning fluorimetry.

Purified RefZ or rLOF variants from frozen stocks (50 mM Tris-HCl [pH 9.0], 300 mM KCl, 10% [vol/vol] glycerol, and 250 mM imidazole) were thawed and diluted in 20 mM Tris-HCl (pH 7.5) to a final concentration of 10 μM. To ensure identical final concentrations of storage buffer for all rLOF variants, reaction mixtures were normalized to the maximum required concentration of storage buffer determined by the lowest rLOF variant concentration; the final buffer concentration was 0.16×. All reaction mixtures contained 5× Sypro orange protein gel stain (Thermo Fisher) diluted to a working concentration in DMSO. The DSF assays were performed in a 96-well hard-shell PCR plate (Bio-Rad; HSP9601) using a CFX96 Touch real-time PCR detection system (Bio-Rad). The reactions were ramped from 25°C to 95°C at a rate of 1°C min^−1^.

### Data availability.

The data that support the findings of this study are found within the article and supplemental material or are available upon reasonable request from the corresponding author (J. K. Herman). The coordinates and structure factors for RefZ have been deposited in the Protein Data Bank (PDB ID 6MJ1).

## Supplementary Material

Supplemental file 1
