# Supplementary material for: A DNA-Binding Protein Tunes Septum Placement during Bacillus subtilis Sporulation
Source: J Bacteriol. 2019 Jul 24;201(16):e00287-19. doi: 10.1128/JB.00287-19 (PMC6657595; doi:10.1128/JB.00287-19)
Supplement: Supplemental file 1 [file JB.00287-19-s0001.pdf]

Supplementary materials

A DNA-binding protein tunes septum placement during *Bacillus subtilis* sporulation

Emily E. Brown[1,%], Allyssa K. Miller[1,%], Inna V. Krieger[2], Ryan M. Otto[1], James C. Sacchettini[1,2] and Jennifer K. Herman[1*]

Department of Biochemistry and Biophysics, Texas A&M University, College Station, TX USA

1Department of Biochemistry and Biophysics, Texas A&M University, College Station TX

2Department of Chemistry, Texas A&M University, College Station TX

[%]Authors contributed equally

*Corresponding author

Short title: Septum positioning during *B. subtilis* sporulation

Text S1. Strain and plasmid construction
Table S1. Strains used in this study
Table S2. Plasmids
Table S3. Oligonucleotides
Table S4. Polymorphisms identified in the rLOF selection-screen
Table S5. Data collection, phasing and refinement statistics for the RefZ structure
Figure S1. Superimposition of the N-terminal domains of RefZ and QacR.
Figure S2. Example purification profiles of wild-type RefZ and rLOF variants.
Figure S3. EMSA laddering behavior of wild-type RefZ and rLOF variants.
Figure S4. Size-exclusion chromatogram for WT RefZ.
Figure S5. Bacterial two-hybrid assay spot plate replicas of RefZ and rLOF self-interaction.
Figure S6. Thermostability of RefZ and the rLOF variants.
Figure S7. Bacterial two-hybrid assay for RefZ and FtsZ.

Text S1. Strain and plasmid construction

Strain construction (in alpha-numerical order)

Solid medium plates used for selections were made from lysogeny broth (LB, Lennox) with 1.5% (w/v) Bacto™ agar supplemented with the indicated concentrations of antibiotics/supplements. Integration into the *amyE* locus was assayed for by loss of amylase activity following growth on LB plates supplemented with 1% (w/v) soluble potato starch (EMD) and overlayed with Gram's Iodine (Ricca Chemical Company). Where appropriate, transformants were screened for parental background resistances and on LB plates supplemented with 40 µg ml^{-1} X-gal to visually screen for *lacZ* expression from the P_{spremo} promoter.

BAM043 was created by transformation of *B. subtilis* 168 with genomic DNA isolated from BJH042 selecting for *minD* deletion on 10 µg ml^{-1} kanamycin plates.

BAM075 was created by transformation of *B. subtilis* 168 with linearized pDR111 (P_{hy}-*empty*), selecting for integration at the *amyE* locus on 100 µg ml^{-1} spectinomycin plates.

BAM110 was created by transformation of BJH294 with pJW004 selecting for integration of P_{hy}-*empty* at the *yhdG* locus on 0.8 µg ml^{-1} phleomycin plates and patched on 7.5 µg ml^{-1} chloramphenicol plates to confirm loss of parental resistance.

BAM111 was created by transformation of BJH294 with pJW014 selecting for integration of P_{hy}-*refZ* at the *yhdG* locus on 0.8 µg ml^{-1} phleomycin plates and patched on 7.5 µg ml^{-1} chloramphenicol plates to confirm loss of parental resistance.

BAM142 was created by transformation of *B. subtilis* 168 with genomic DNA isolated from BJH188 selecting for integration of P_{xylA}-*comK* at the *lacA* locus on 1 µg ml^{-1} erythromycin (erm) plus 25 µg ml^{-1} lincomycin (MLS) plates.

BAM168 (selection-screen background) was created by transformation of BAM266 with genomic DNA isolated from BAM043 (*minD::kan)* selecting for integration on 10 µg ml^{-1} kanamycin plates.

BAM229 was created by transformation of *B. subtilis* 168 with linearized pAM083 (Plasmid Construction), selecting for P_{spremo}-*lacZ* integration at the *sacA* locus on 7.5 µg ml^{-1} chloramphenicol plates. The *sacA* locus was screened for size by PCR with OAM124 and OAM125. PCR products of the expected size were sequenced with OJH133 to confirm promoter fusion.

BAM248 was created by transformation of BAM229 with genomic DNA isolated from BJH247 (*refZ::tet*) selecting for integration on 10 µg ml^{-1} tetracycline plates.

BAM266 was created by transformation of BAM248 with genomic DNA isolated from BAM142 selecting for integration of P_{xylA}-*comK* at the *lacA* locus on 1 µg ml^{-1} erythromycin (erm) plus 25 µg ml^{-1} lincomycin (MLS) plates.

BAM374 (P_{hy}-*refZ* in selection-screen background) was created by transformation of super-competent BAM168 cells with genomic DNA isolated from BJH228 (pJW013 integrated at *amyE*) selecting for integration of P_{hy}-*refZ* at the *amyE* locus on 100 µg ml^{-1} spectinomycin

plates supplemented with 0.2% (v/v) glucose to repress leaky expression from the P_{hy} promoter (even moderate expression of wildtype *refZ* produces a growth defect in a Δ*minD* background).

BAM390 (P_{hy}-*empty* in the selection-screen background) was created by transformation of super-competent BAM168 cells with genomic DNA isolated from BAM075 (pDR111 integrated at *amyE*) selecting for integration of P_{hy}-*empty* at the *amyE* locus on 100 µg ml^{-1} spectinomycin plates.

BAM400, 403, 407, 409, 411, 440, 443, 444, 449, 462 (P_{hy}-*rLOF* mutants in clean selection-screen background) were created similar to BAM374, except genomic DNA prepared from the original *rLOF* mutant strains (BAM1060-1069) was transformed into super-competent BAM168 cells selecting for integration of P_{hy}-*rLOF* at the *amyE* locus on 100 µg ml^{-1} spectinomycin plates supplemented with 0.2% (v/v) glucose.

BAM428, 431, 434, 436, 450, 451, 454, 455, 457, 490 (P_{hy}-*rLOF* in wildtype background) were created by transformation of *B. subtilis* 168 with genomic DNA prepared from the original *rLOF* mutant strains (BAM1060-1069) selecting for integration of P_{hy}-*rLOF* at the *amyE* locus on 100 µg ml^{-1} spectinomycin plates.

BAM1006-BAM1027 (P_{refZ}-*refZ* and P_{refZ}-*rLOF* Reporter Trapping Strains) were created by transformation of BJH245 and BAM078 (the left and right arm reporter backgrounds, respectively) with linear DNA constructs [UP_{refZ} + P_{refZ}-*rLOF* (or P_{refZ}-*refZ*) + *(cat^R)* + $DOWN_{refZ}$] generated by assembly(1) of the following DNA fragments:

<u>UP_{refZ}</u>
The upstream chromosomal region flanking the *refZ* gene, including the native promoter, was amplified by PCR with OAM200 and OAM201 from genomic DNA prepared from *B. subtilis* 168, to create a large region of homology for double crossover integration at the native *refZ* locus.

<u>P_{refZ}-*rLOF* (or P_{refZ}-*refZ*)</u>
The wildtype *refZ* sequence and the 10 *rLOF* mutant sequences were amplified by PCR with OAM202 and OAM203 from genomic DNA prepared from BJH228 (pJW013 integrated at *amyE*) and BAM1060-1069 (original *rLOF* mutant strains), respectively. PCR reactions were resolved on 0.8% agarose gels and purified following extraction. OAM202 introduces 27 bp to the 5' end with homology to the 3' end of the "UP_{refZ}" fragment (see above). OAM203 introduces 24 bp to the 3' end with homology to the 5' end of the "*(cat^R)*" fragment (below).

<u>*(cat^R)*</u>
A chloramphenicol resistance gene (*cat^R*) and its associated promoter were amplified by PCR with OJH179 and OJH180 from plasmid pKM074, to provide a selectable marker for assembly integration of the assembled construct at the native *refZ* locus.

<u>$DOWN_{refZ}$</u>
The downstream chromosomal region flanking the *refZ* gene was amplified by PCR with OAM204 and OAM205 from genomic DNA prepared from *B. subtilis* 168, to create a large region of homology for double crossover integration at the native *refZ* locus. OAM204 introduces homology to the 3' end of the "*(cat^R)*" fragment.

Assembly reactions (20 µl each) were transformed into 0.2 ml of competent cells with selection for integration at the native *refZ* locus on 7.5 µg ml^{-1} chloramphenicol plates. Genomic DNA was isolated and *refZ* chromosomal regions were screened for size by PCR with OAM200 and

OAM205. Fragments of expected size were sequenced with OEB041 or OEB042 to confirm the presence of the *rLOF* mutation.

BAM1060-1069 (P_{hy}-*rLOF* mutants obtained in the selection-screen) were isolated following transformation of super-competent BAM168 cells with linear DNA constructs [UP_{amyE}-($spec^R$)-P_{hy} + *refZ** + *lacI*-$DOWN_{amyE}$] generated by Gibson assembly(1) of the following DNA fragments:

<u>UP_{amyE}-($spec^R$)-P_{hy}</u>
The upstream chromosomal region flanking the *amyE* gene, the spectinomycin resistance gene and its associated promoter, and the P_{hy} promoter were amplified by PCR with OAM010 and OAM013 from genomic DNA isolated from BJH228 (pJW013 integrated at *amyE*).

<u>*refZ**</u>
refZ open-reading frame was PCR amplified from pJW013 with Phusion High-Fidelity polymerase using OAM122 and OAM165 to create the template for mutagenesis. The resulting template was mutagenized by error-prone PCR with OAM122 and OAM166 using the GeneMorph II Random Mutagenesis Kit according to the manufacturer's protocol (Agilent Technologies #200550) to generate a pool of mutant *refZ* PCR fragments (*refZ**). OAM122 introduces 5' sequence homology to the P_{hy} promoter (see above) and OAM166 introduces 3' sequence homology to the *lacI*-$DOWN_{amyE}$ fragment.

<u>*lacI*-$DOWN_{amyE}$</u>
The *lacI* repressor gene and the downstream chromosomal region flanking the *amyE* gene were amplified by PCR with OAM001 and OAM012 from genomic DNA isolated from BJH228 (pJW013 integrated at *amyE*).

Assembly reactions were placed on ice and transformed directly into super-competent BAM168, selecting for integration at *amyE* on 100 µg ml^{-1} spectinomycin plates supplemented with 1 mM IPTG. Super-competent BAM168 cells were prepared and transformed as described in *Selection of rLOF mutants* (Experimental Procedures).

BAM1662 was created by transformation of BAM390 with genomic DNA prepared from BAM110 selecting for integration at *yhdG* on 0.8 µg ml^{-1} phleomycin plates supplemented with 0.2% glucose at 30°C overnight.

BAM1665 was created by transformation of BAM374 with genomic DNA prepared from BAM110 selecting for integration at *yhdG* on 0.8 µg ml^{-1} phleomycin plates supplemented with 0.2% glucose at 30°C overnight.

BAM1664-1676 were created by transformation of the corresponding P_{hy}-*rLOF* mutants in a clean selection-screen background (BAM374, 390, 400, 403, 407, 409, 411, 440, 443, 444, 449, 462) with genomic DNA prepared from BAM111 selecting for integration at *yhdG* on 0.8 µg ml^{-1} phleomycin plates supplemented with 0.2% glucose at 30°C overnight.

BJH042 was created by transformation of BDR2353 (*minD::kan*) with linearized pJK013 selecting for integration of P_{hy}-*refZ* at the *amyE* locus on 100 µg ml^{-1} spectinomycin plates.

BJH228 was created by transformation of *B. subtilis* 168 with genomic DNA isolated from BJW123 selecting for integration of P_{hy}-*refZ* at the *amyE* locus on 100 µg ml^{-1} spectinomycin plates.

BJH294 was created by transformation of *B. subtilis* 168 with genomic DNA isolated from BDR2260 selecting for integration of the chloramphenicol resistance gene at the *yhdG* locus on 5 µg ml^{-1} chloramphenicol plates. BDR2260 contains plasmid pBB275 [*yhdG::cat (amp)*], an ectopic integration vector for double crossover insertions into the *yhdG* locus (B. Burton and D.Z. Rudner, unpublished).

Plasmid construction

Plasmid sequences are available upon request.

pAM037 [*yycR::*P_{spremo} *(cat)*] was generated by cloning the annealed product of oligos OAM139 and OAM140 into pJW034 between XhoI-HindIII.

pAM046 [*sacA::(cat)*] was generated by subcloning the *cat* (chloramphenicol) resistance cassette from pKM074 into the backbone of pKM062 between SalI-BamHI.

pAM080 [*sacA::*P_{spremo} *(cat)*] was generated by cloning PCR product of OJH133 and OJH001 amplified from pAM037 into pAM046 between EcoRI-HindIII.

pAM083 [*sacA::*P_{spremo}*-lacZ (cat)*] was generated by cloning PCR product of OJH185 and OJH186 amplified from pJH036 into pAM080 between HindIII-NheI.

pAM139 ($RefZ^{Y43A}$-T25) was generated by cloning the PCR product of OAM148 and OAM149 amplified from pRD010 into pKNT25 (empty-T25 plasmid) between SphI-BamHI. Plasmids were confirmed by PCR with OYD070 and OAM149 and products were sequenced to confirm the presence of the *rLOF* mutation.

pAM141 ($RefZ^{R106A}$-T25) was generated by cloning the PCR product of OAM148 and OAM149 amplified from pRD001 into pKNT25 (empty-T25 plasmid) between SphI-BamHI. Plasmids were confirmed by PCR with OYD070 and OAM149 and products were sequenced to confirm the presence of the *rLOF* mutation.

pAM144 ($RefZ^{Y43A}$-T18) was generated by cloning the PCR product of OAM148 and OAM149 amplified from pRD010 into pCH363 (empty-T18 plasmid) between SphI-BamHI. Plasmids were confirmed by PCR with OYD070 and OAM149 and products were sequenced to confirm the presence of the *rLOF* mutation.

pAM146 ($RefZ^{R106A}$-T18) was generated by cloning the PCR product of OAM148 and OAM149 amplified from pRD001 into pCH363 (empty-T18 plasmid) between SphI-BamHI. Plasmids were confirmed by PCR with OYD070 and OAM149 and products were sequenced to confirm the presence of the *rLOF* mutation.

pAM152-161 (rLOF-T18 B2H plasmids) were generated by cloning the PCR products of OAM148 and OAM149 from genomic DNA prepared from corresponding left arm rLOF Reporter Trapping strains (BAM1006-1026, even numbered strains) into pCH363 (empty-T18 plasmid) between SphI-BamHI. Plasmids were confirmed by PCR with OYD070 and OAM149 and products were sequenced to confirm the presence of the *rLOF* mutations.

pAM162-171 (rLOF-T25 B2H plasmids) were generated by cloning the PCR products of OAM148 and OAM149 from genomic DNA prepared from corresponding left arm rLOF Reporter Trapping strains (BAM1006-1026, even numbered strains) into pKNT25 (empty-T25 plasmid) between SphI-BamHI. Plasmids were confirmed by PCR with OYD070 and OAM149 and products were sequenced to confirm the presence of the *rLOF* mutations.

pEB013 (RefZ^{R116S}-His6) was generated by cloning the PCR product from OEB041 and OEB042 amplification of genomic DNA from BAM1064 into pET-24b (+) (NheI and XhoI). Confirmed by sequencing.

pEB014 (RefZ^{E117G}-His6) was generated by cloning PCR product from OEB041 and OEB042 amplification of genomic DNA from BAM1067 into pET-24b (+) (NheI and XhoI). Confirmed by sequencing.

pEB015 (RefZ^{E117D}-His6) was generated by cloning PCR product from OEB041 and OEB042 amplification of genomic DNA from BAM1066 into pET-24b (+) (NheI and XhoI). Confirmed by sequencing.

pEB016 (RefZ^{E179K}-His6) was generated by cloning PCR product from OEB041 and OEB042 amplification of genomic DNA from BAM1069 into pET-24b (+) (NheI and XhoI). Confirmed by sequencing.

pEB017 (RefZ^{R102C}-His6) was generated by cloning PCR product from OEB041 and OEB042 amplification of genomic DNA from BAM1062 into pET-24b (+) (NheI and XhoI). Confirmed by sequencing.

pEB018 (RefZ^{R102S}-His6) generated by cloning PCR product from OEB041 and OEB042 amplification of genomic DNA from BAM1063 into pET-24b (+) (NheI and XhoI). Confirmed by sequencing.

pEB019 (RefZ^{E53K}-His6) generated by cloning PCR product from OEB041 and OEB042 amplification of genomic DNA from BAM1060 into pET-24b (+) (NheI and XhoI). Confirmed by sequencing.

pEB020 (RefZ^{E61K}-His6) was generated by cloning PCR product from OEB041 and OEB042 amplification of genomic DNA from BAM1061 into pET-24b (+) (NheI and XhoI). Confirmed by sequencing.

pEB021 (RefZ^{L153R}-His6) was generated by cloning PCR product from OEB041 and OEB042 amplification of genomic DNA from BAM1068 into pET-24b (+) (NheI and XhoI). Confirmed by sequencing.

pEB022 (RefZ^{R116W}-His6) was generated by cloning PCR product from OEB041 and OEB042 amplification of genomic DNA from BAM1065 into pET-24b (+) (NheI and XhoI). Confirmed by sequencing.

pJW096 (RefZ-T25) was generated by cloning PCR product from OJW167 and OJW168 amplification of genomic DNA from *B. subtilis* wild-type PY79 into pKNT25 (SphI and BamHI). Confirmed by restriction enzyme digestion.

pJW097 (RefZ-T18) was generated by cloning PCR product from OJW167 and OJW168 amplification of genomic DNA from *B. subtilis* wild-type PY79 into pCH363 (SphI and BamHI). Confirmed by restriction enzyme digestion.

pJW098 (FtsZ-T25) was generated by cloning PCR product from OJW169 and OJW170 amplification of genomic DNA from *B. subtilis* wild-type PY79 into pKNT25 (SphI and BamHI). Confirmed by restriction enzyme digestion.

pJW099 (FtsZ-T18) was generated by cloning PCR product from OJW169 and OJW170 amplification of genomic DNA from *B. subtilis* wild-type PY79 into pCH363 (SphI and BamHI). Confirmed by restriction enzyme digestion.

pJW100 (T25-RefZ) was generated by cloning PCR product from OJW171 and OJW172 amplification of genomic DNA from *B. subtilis* wild-type PY79 into pKT25 (EcoRI and BamHI). Confirmed by restriction enzyme digestion.

pJW101 (T18-RefZ) was generated by cloning PCR product from OJW171 and OJW172 amplification of genomic DNA from *B. subtilis* wild-type PY79 into pCH364 (EcoRI and BamHI). Confirmed by restriction enzyme digestion.

pJW102 (T25-FtsZ) was generated by cloning PCR product from OJW173 and OJW174 amplification of genomic DNA from *B. subtilis* wild-type PY79 into pKT25 (EcoRI and BamHI). Confirmed by restriction enzyme digestion.

pJW103 (T18-FtsZ) was generated by cloning PCR product from OJW173 and OJW174 amplification of genomic DNA from *B. subtilis* wild-type PY79 into pCH364 (EcoRI and BamHI). Confirmed by restriction enzyme digestion.

pLM025 ($RefZ^{WT}$-His6) was generated by cloning PCR product from OLM048 and OLM049 amplification of genomic DNA from PY79 into pET-24b (+) (NheI and XhoI). Confirmed by sequencing.

Table S1. Strains used in this study

**Strain**	**Description**	**Reference**
B. subtilis 168	*Bacillus subtilis* laboratory strain 168 *trpC2*	Bacillus Genetic Stock Center (1A866)
BL21 (DE3)	*BL21 (DE3) pLysS (cat)*	Expression host
DH5α	*F-, endA1, glnV44, thi-1, recA1, relA1, gyrA96, deoR, nupG, Φ80dlacZΔM15, Δ(lacZYA-argF)U169, hsdR17(*r_K^- m_K^+*), λ–*	
DHP1	*F-, cya-99, araD139, galE15, galK16, rpsL1 (Strr), hsdR2, mcrA1,mcrB1*	Obtained from Thomas Bernhardt
B. subtilis subsp. subtilis		
BJH188	*Em his nprE18 aprE3 eglS(DELTA)102 bglT/bglS(DELTA)EV lacA::*P_{xylA}*-comK (ERM)*	Bacillus Genetic Stock Center (1A976)
**PY79**		
BDR2260	*yhdG::cat*	David Rudner
BDR2353	*minD::kan*	David Rudner

BJH042	*minD::kan, amyE::P_{hy}-refZ (spec)*	This work
BJW123	*amyE::P_{hy}-empty (spec)*	(2)
B. subtilis 168		
BAM043	*minD::kan*	This work
BAM075	*amyE::P_{hy}-empty (spec)*	This work
BAM077	*RBM_{5mu}, yycR(-7°)::P_{spoIIQ}-yfp (phleo), +51°::P_{spoIIQ}-cfp (erm), spoIIIE36-tet*	(3)
BAM078	*yycR(-7°)::P_{spoIIQ}-yfp (phleo), +51°::P_{spoIIQ}-cfp (erm), spoIIIE36-tet*	(3)
BAM079	*refZ::cat, yycR(-7°)::P_{spoIIQ}-yfp (phleo), +51°::P_{spoIIQ}-cfp (erm), spoIIIE36-tet*	(3)
BAM110	*yhdG::P_{hy}-empty (phleo)*	This work
BAM111	*yhdG::P_{hy}-refZ (WT) (phleo)*	This work
BAM142	*lacA::P_{xylA}-comK (erm)*	This work
BAM168	*sacA::P_{spremo}-lacZ (cat), refZ::tet, lacA::P_{xylA}-comK (erm), minD::kan*	This work
BAM229	*sacA::P_{spremo}-lacZ (cat)*	This work
BAM248	*sacA::P_{spremo}-lacZ (cat), refZ::tet*	This work
BAM266	*sacA::P_{spremo}-lacZ (cat), refZ::tet, lacA::P_{xylA}-comK (erm)*	This work
BAM374	*sacA::P_{spremo}-lacZ (cat), refZ::tet, lacA::P_{xylA}-comK (erm), minD::kan, amyE::P_{hy}-refZ (spec)*	Fig 1B
BAM390	*sacA::P_{spremo}-lacZ (cat), refZ::tet, lacA::P_{xylA}-comK (erm), minD::kan, amyE::P_{hy}-empty (spec)*	Fig 1B
BAM400	*sacA::P_{spremo}-lacZ (cat), refZ::tet, lacA::P_{xylA}-comK (erm), minD::kan, amyE::P_{hy}-refZ (E179K) (spec)*	Fig 1B
BAM403	*sacA::P_{spremo}-lacZ (cat), refZ::tet, lacA::P_{xylA}-comK (erm), minD::kan, amyE::P_{hy}-refZ (E117G) (spec)*	Fig 1B
BAM407	*sacA::P_{spremo}-lacZ (cat), refZ::tet, lacA::P_{xylA}-comK (erm), minD::kan, amyE::P_{hy}-refZ (R102C) (spec)*	Fig 1B
BAM409	*sacA::P_{spremo}-lacZ (cat), refZ::tet, lacA::P_{xylA}-comK (erm), minD::kan, amyE::P_{hy}-refZ (R102S) (spec)*	Fig 1B
BAM411	*sacA::P_{spremo}-lacZ (cat), refZ::tet, lacA::P_{xylA}-comK (erm), minD::kan, amyE::P_{hy}-refZ (L153R) (spec)*	Fig 1B
BAM428	*amyE::P_{hy}-refZ (E179K) (spec)*	Fig 1C & D
BAM431	*amyE::P_{hy}-refZ (E117G) (spec)*	Fig 1C & D
BAM434	*amyE::P_{hy}-refZ (R102C) (spec)*	Fig 1C & D
BAM436	*amyE::P_{hy}-refZ (R102S) (spec)*	Fig 1C & D
BAM440	*sacA::P_{spremo}-lacZ (cat), refZ::tet, lacA::P_{xylA}-comK (erm), minD::kan, amyE::P_{hy}-refZ (R116W) (spec)*	Fig 1B
BAM443	*sacA::P_{spremo}-lacZ (cat), refZ::tet, lacA::P_{xylA}-comK (erm), minD::kan, amyE::P_{hy}-refZ (R116S) (spec)*	Fig 1B
BAM444	*sacA::P_{spremo}-lacZ (cat), refZ::tet, lacA::P_{xylA}-comK (erm), minD::kan, amyE::P_{hy}-refZ (E117D) (spec)*	Fig 1B
BAM449	*sacA::P_{spremo}-lacZ (cat), refZ::tet, lacA::P_{xylA}-comK (erm), minD::kan, amyE::P_{hy}-refZ (E53K) (spec)*	Fig 1B
BAM450	*amyE::P_{hy}-refZ (L153R) (spec)*	Fig 1C & D
BAM451	*amyE::P_{hy}-refZ (R116W) (spec)*	Fig 1C & D

BAM454	*amyE::*P_{hy}*-refZ (R116S) (spec)*	Fig 1C & D
BAM455	*amyE::*P_{hy}*-refZ (E117D) (spec)*	Fig 1C & D
BAM457	*amyE::*P_{hy}*-refZ (E53K) (spec)*	Fig 1C & D
BAM462	*sacA::*P_{spremo}*-lacZ (cat), refZ::tet, lacA::*P_{xylA}*-comK (erm), minD::kan, amyE::*P_{hy}*-refZ (E61K) (spec)*	Fig 1B
BAM490	*amyE::*P_{hy}*-refZ (E61K) (spec)*	Fig 1C & D
BAM1006	*refZ::refZ (WT) (cat), yycR(-7°)::*P_{spoIIQ}*-yfp (phleo), lacA(-61°)::*P_{spoIIQ}*-cfp (erm), spoIIIE36-tet*	Fig 2B
BAM1007	*refZ::refZ (WT) (cat), yycR(-7°)::*P_{spoIIQ}*-yfp (phleo), +51°::*P_{spoIIQ}*-cfp (erm), spoIIIE36-tet*	Fig 2B
BAM1008	*refZ::refZ (E179K) (cat), yycR(-7°)::*P_{spoIIQ}*-yfp (phleo), lacA(-61°)::*P_{spoIIQ}*-cfp (erm), spoIIIE36-tet*	Fig 2B
BAM1009	*refZ::refZ (E179K) (cat), yycR(-7°)::*P_{spoIIQ}*-yfp (phleo), +51°::*P_{spoIIQ}*-cfp (erm), spoIIIE36-tet*	Fig 2B
BAM1010	*refZ::refZ (E117G) (cat), yycR(-7°)::*P_{spoIIQ}*-yfp (phleo), lacA(-61°)::*P_{spoIIQ}*-cfp (erm), spoIIIE36-tet*	Fig 2B
BAM1011	*refZ::refZ (E117G) (cat), yycR(-7°)::*P_{spoIIQ}*-yfp (phleo), +51°::*P_{spoIIQ}*-cfp (erm), spoIIIE36-tet*	Fig 2B
BAM1012	*refZ::refZ (R012C) (cat), yycR(-7°)::*P_{spoIIQ}*-yfp (phleo), lacA(-61°)::*P_{spoIIQ}*-cfp (erm), spoIIIE36-tet*	Fig 2B
BAM1013	*refZ::refZ (R102C) (cat), yycR(-7°)::*P_{spoIIQ}*-yfp (phleo), +51°::*P_{spoIIQ}*-cfp (erm), spoIIIE36-tet*	Fig 2B
BAM1014	*refZ::refZ (R102S) (cat), yycR(-7°)::*P_{spoIIQ}*-yfp (phleo), lacA(-61°)::*P_{spoIIQ}*-cfp (erm), spoIIIE36-tet*	Fig 2B
BAM1015	*refZ::refZ (R102S) (cat), yycR(-7°)::*P_{spoIIQ}*-yfp (phleo), +51°::*P_{spoIIQ}*-cfp (erm), spoIIIE36-tet*	Fig 2B
BAM1016	*refZ::refZ (L153R) (cat), yycR(-7°)::*P_{spoIIQ}*-yfp (phleo), lacA(-61°)::*P_{spoIIQ}*-cfp (erm), spoIIIE36-tet*	Fig 2B
BAM1017	*refZ::refZ (L153R) (cat), yycR(-7°)::*P_{spoIIQ}*-yfp (phleo), +51°::*P_{spoIIQ}*-cfp (erm), spoIIIE36-tet*	Fig 2B
BAM1018	*refZ::refZ (R116W) (cat), yycR(-7°)::*P_{spoIIQ}*-yfp (phleo), lacA(-61°)::*P_{spoIIQ}*-cfp (erm), spoIIIE36-tet*	Fig 2B
BAM1019	*refZ::refZ (R116W) (cat), yycR(-7°)::*P_{spoIIQ}*-yfp (phleo), +51°::*P_{spoIIQ}*-cfp (erm), spoIIIE36-tet*	Fig 2B
BAM1020	*refZ::refZ (R116S) (cat), yycR(-7°)::*P_{spoIIQ}*-yfp (phleo), lacA(-61°)::*P_{spoIIQ}*-cfp (erm), spoIIIE36-tet*	Fig 2B
BAM1021	*refZ::refZ (R116S) (cat), yycR(-7°)::*P_{spoIIQ}*-yfp (phleo), +51°::*P_{spoIIQ}*-cfp (erm), spoIIIE36-tet*	Fig 2B
BAM1022	*refZ::refZ (E117D) (cat), yycR(-7°)::*P_{spoIIQ}*-yfp (phleo), lacA(-61°)::*P_{spoIIQ}*-cfp (erm), spoIIIE36-tet*	Fig 2B
BAM1023	*refZ::refZ (E117D) (cat), yycR(-7°)::*P_{spoIIQ}*-yfp (phleo), +51°::*P_{spoIIQ}*-cfp (erm), spoIIIE36-tet*	Fig 2B
BAM1024	*refZ::refZ (E53K) (cat), yycR(-7°)::*P_{spoIIQ}*-yfp (phleo), lacA(-61°)::*P_{spoIIQ}*-cfp (erm), spoIIIE36-tet*	Fig 2B
BAM1025	*refZ::refZ (E53K) (cat), yycR(-7°)::*P_{spoIIQ}*-yfp (phleo), +51°::*P_{spoIIQ}*-cfp (erm), spoIIIE36-tet*	Fig 2B
BAM1026	*refZ::refZ (E61K) (cat), yycR(-7°)::*P_{spoIIQ}*-yfp (phleo), lacA(-61°)::*P_{spoIIQ}*-cfp (erm), spoIIIE36-tet*	Fig 2B

BAM1027	*refZ::refZ (E61K) (cat), yycR(-7°)::P_{spoIIQ}-yfp (phleo), +51°::P_{spoIIQ}-cfp (erm), spoIIIE36-tet*	Fig 2B
BAM1060	*sacA::P_{spremo}-lacZ (cat), refZ::tet, lacA::P_{xylA}-comK (erm), minD::kan, amyE::P_{hy}-refZ (E53K) (spec)*	Original rLOF isolate
BAM1061	*sacA::P_{spremo}-lacZ (cat), refZ::tet, lacA::P_{xylA}-comK (erm), minD::kan, amyE::P_{hy}-refZ (E61K) (spec)*	Original rLOF isolate
BAM1062	*sacA::P_{spremo}-lacZ (cat), refZ::tet, lacA::P_{xylA}-comK (erm), minD::kan, amyE::P_{hy}-refZ (R102C) (spec)*	Original rLOF isolate
BAM1063	*sacA::P_{spremo}-lacZ (cat), refZ::tet, lacA::P_{xylA}-comK (erm), minD::kan, amyE::P_{hy}-refZ (R102S) (spec)*	Original rLOF isolate
BAM1064	*sacA::P_{spremo}-lacZ (cat), refZ::tet, lacA::P_{xylA}-comK (erm), minD::kan, amyE::P_{hy}-refZ (R116S) (spec)*	Original rLOF isolate
BAM1065	*sacA::P_{spremo}-lacZ (cat), refZ::tet, lacA::P_{xylA}-comK (erm), minD::kan, amyE::P_{hy}-refZ (R116W) (spec)*	Original rLOF isolate
BAM1066	*sacA::P_{spremo}-lacZ (cat), refZ::tet, lacA::P_{xylA}-comK (erm), minD::kan, amyE::P_{hy}-refZ (E117D) (spec)*	Original rLOF isolate
BAM1067	*sacA::P_{spremo}-lacZ (cat), refZ::tet, lacA::P_{xylA}-comK (erm), minD::kan, amyE::P_{hy}-refZ (E117G) (spec)*	Original rLOF isolate
BAM1068	*sacA::P_{spremo}-lacZ (cat), refZ::tet, lacA::P_{xylA}-comK (erm), minD::kan, amyE::P_{hy}-refZ (L153R) (spec)*	Original rLOF isolate
BAM1069	*sacA::P_{spremo}-lacZ (cat), refZ::tet, lacA::P_{xylA}-comK (erm), minD::kan, amyE::P_{hy}-refZ (E179K) (spec)*	Original rLOF isolate
BAM1662	*sacA::P_{spremo}-lacZ (cat), refZ::tet, lacA::P_{xylA}-comK (erm), minD::kan, amyE::P_{hy}-empty (spec), yhdG::P_{hy}-empty (phleo)*	Fig 2A
BAM1664	*sacA::P_{spremo}-lacZ (cat), refZ::tet, lacA::P_{xylA}-comK (erm), minD::kan, amyE::P_{hy}-empty (spec), yhdG::P_{hy}-refZ (WT) (phleo)*	Fig 2A
BAM1665	*sacA::P_{spremo}-lacZ (cat), refZ::tet, lacA::P_{xylA}-comK (erm), minD::kan, amyE::P_{hy}-refZ (WT)(spec), yhdG::P_{hy}-empty (phleo)*	Fig 2A
BAM1666	*sacA::P_{spremo}-lacZ (cat), refZ::tet, lacA::P_{xylA}-comK (erm), minD::kan, amyE::P_{hy}-refZ (WT)(spec), yhdG::P_{hy}-refZ (WT) (phleo)*	Fig 2A
BAM1667	*sacA::P_{spremo}-lacZ (cat), refZ::tet, lacA::P_{xylA}-comK (erm), minD::kan, amyE::P_{hy}-refZ (E53K)(spec), yhdG::P_{hy}-refZ (WT) (phleo)*	Fig 2A
BAM1668	*sacA::P_{spremo}-lacZ (cat), refZ::tet, lacA::P_{xylA}-comK (erm), minD::kan, amyE::P_{hy}-refZ (E61K)(spec), yhdG::P_{hy}-refZ (WT) (phleo)*	Fig 2A
BAM1669	*sacA::P_{spremo}-lacZ (cat), refZ::tet, lacA::P_{xylA}-comK (erm), minD::kan, amyE::P_{hy}-refZ (R102C)(spec), yhdG::P_{hy}-refZ (WT) (phleo)*	Fig 2A
BAM1670	*sacA::P_{spremo}-lacZ (cat), refZ::tet, lacA::P_{xylA}-comK (erm), minD::kan, amyE::P_{hy}-refZ (R102S)(spec), yhdG::P_{hy}-refZ (WT) (phleo)*	Fig 2A
BAM1671	*sacA::P_{spremo}-lacZ (cat), refZ::tet, lacA::P_{xylA}-comK (erm), minD::kan, amyE::P_{hy}-refZ (R116S)(spec), yhdG::P_{hy}-refZ (WT) (phleo)*	Fig 2A
BAM1672	*sacA::P_{spremo}-lacZ (cat), refZ::tet, lacA::P_{xylA}-comK (erm), minD::kan, amyE::P_{hy}-refZ (R116W)(spec), yhdG::P_{hy}-refZ (WT) (phleo)*	Fig 2A
BAM1673	*sacA::P_{spremo}-lacZ (cat), refZ::tet, lacA::P_{xylA}-comK (erm), minD::kan, amyE::P_{hy}-refZ (E117D)(spec), yhdG::P_{hy}-refZ (WT) (phleo)*	Fig 2A
BAM1674	*sacA::P_{spremo}-lacZ (cat), refZ::tet, lacA::P_{xylA}-comK (erm), minD::kan, amyE::P_{hy}-refZ (E117G)(spec), yhdG::P_{hy}-refZ (WT) (phleo)*	Fig 2A
BAM1675	*sacA::P_{spremo}-lacZ (cat), refZ::tet, lacA::P_{xylA}-comK (erm), minD::kan, amyE::P_{hy}-refZ (L153R)(spec), yhdG::P_{hy}-refZ (WT) (phleo)*	Fig 2A

BAM1676	*sacA::P_{spremo}-lacZ (cat), refZ::tet, lacA::P_{xylA}-comK (erm), minD::kan, amyE::P_{hy}-refZ (E179K)(spec), yhdG::P_{hy}-refZ (WT) (phleo)*	Fig 2A
BJH205	*RBM_{5mu}*	(3)
BJH228	*amyE::P_{hy}-refZ (spec)*	Fig 1C & D
BJH245	*yycR(-7°)::P_{spoIIQ}-yfp (phleo), lacA(-61°)::P_{spoIIQ}-cfp (erm), spoIIIE36-tet*	(3)
BJH246	*RBM_{5mu}, yycR(-7°)::P_{spoIIQ}-yfp (phleo), lacA(-61°)::P_{spoIIQ}-cfp (erm), spoIIIE36-tet*	(3)
BJH251	*refZ::cat, yycR(-7°)::P_{spoIIQ}-yfp (phleo), lacA(-61°)::P_{spoIIQ}-cfp (erm), spoIIIE36-tet*	(3)
BJH294	*yhdG::cat*	This work

Table S2. Plasmids

**Plasmid**	**Description**	**Reference**
pAM037	*yycR::P_{spremo} (cat)(amp)*	This work
pAM046	*sacA::cat (amp)*	This work
pAM080	*sacA::P_{spremo} (cat)(amp)*	This work
pAM083	*sacA::P_{spremo}-lacZ (cat)(amp)*	This work
pAM139	*refZ(Y43A)-T25 (kan)*	This work
pAM141	*refZ(R106A)-T25 (kan)*	This work
pAM144	*refZ(Y43A)-T18 (amp)*	This work
pAM146	*refZ(R106A)-T18 (amp)*	This work
pAM152	*refZ(E53K)-T18 (amp)*	This work
pAM153	*refZ(E61K)-T18 (amp)*	This work
pAM154	*refZ(R102C)-T18 (amp)*	This work
pAM155	*refZ(R102S)-T18 (amp)*	This work
pAM156	*refZ(R116S)-T18 (amp)*	This work
pAM157	*refZ(R116W)-T18 (amp)*	This work
pAM158	*refZ(E117D)-T18 (amp)*	This work
pAM159	*refZ(E117G)-T18 (amp)*	This work
pAM160	*refZ(L153R)-T18 (amp)*	This work
pAM161	*refZ(E179K)-T18 (amp)*	This work
pAM162	*refZ(E53K)-T25 (kan)*	This work
pAM163	*refZ(E61K)-T25 (kan)*	This work
pAM164	*refZ(R102C)-T25 (kan)*	This work
pAM165	*refZ(R102S)-T25 (kan)*	This work
pAM166	*refZ(R116S)-T25 (kan)*	This work
pAM167	*refZ(R116W)-T25 (kan)*	This work
pAM168	*refZ(E117D)-T25 (kan)*	This work
pAM169	*refZ(E117G)-T25 (kan)*	This work
pAM170	*refZ(L153R)-T25 (kan)*	This work

pAM171	*refZ(E179K)-T25 (kan)*	This work
pCH363	*empty-T18 (amp)*	Tom Bernhardt
pDR111	*amyE::*P_{hy} *(spec)(amp)*	David Rudner
pEB013	*refZ(R116S)-His6 (kan)*	This work
pEB014	*refZ(E117G)-His6 (kan)*	This work
pEB015	*refZ(E117D)-His6 (kan)*	This work
pEB016	*refZ(E179K)-His6 (kan)*	This work
pEB017	*refZ(R102C)-His6 (kan)*	This work
pEB018	*refZ(R102S)-His6 (kan)*	This work
pEB019	*refZ(E53K)-His6 (kan)*	This work
pEB020	*refZ(E61K)-His6 (kan)*	This work
pEB021	*refZ(L153R)-His6 (kan)*	This work
pEB022	*refZ(R116W)-His6 (kan)*	This work
pET24b (+)	*C-terminal His6-tag*	
pJH036	*sacA::*P_{hy}*-lacZ (erm)(amp)*	This work
pJK013	*amyE::*P_{hy}*-refZ (spec)(amp)*	(2)
pJW004	*yhdG::*P_{hy}*-empty (phleo)(amp)*	(2)
pJW014	*yhdG::*P_{hy}*-refZ (WT) (phleo)(amp)*	(2)
pJW034	*yycR::*P_{hy} *(cat)(amp)*	This work
pJW096	*refZ(WT)-T25 (kan)*	This work
pJW097	*refZ(WT)-T18 (amp)*	(3)
pJW098	*ftsZ-T25 (kan)*	This work
pJW099	*ftsZ-T18 (kan)*	This work
pJW100	*T25-refZ (WT) (kan)*	This work
pJW101	*T18-refZ (WT) (kan)*	This work
pJW102	*T25-ftsZ (kan)*	This work
pJW103	*T25-ftsZ (kan)*	This work
pKM062	*sacA::erm (amp)*	David Rudner
pKM074	*MCS1+2 (cat)(amp)*	David Rudner
pKNT25	*empty-T25 (kan)*	Tom Bernhardt
pLM025	*refZ(WT)-His6 (kan)*	David Rudner
pRD001	*amyE::*P_{hy}*-refZ(R106A) (spec)(amp)*	David Rudner
pRD010	*amyE::*P_{hy}*-refZ(Y43A) (spec)(amp)*	(3)

Table S3. Oligonucleotides

Oligo	Sequence 5' to 3'
OAM001	AGAAGCGTTAGCGGCAGCAAGTGAT
OAM010	ATGGACACAACAACAGCAAAACAGGC

OAM012	GCTAGCCGCATGCAAGCTAATT
OAM013	AGTAGTTCCTCCTTATGTAAGC
OAM122	ATTAAGCTTACATAAGGAGGAACTACTATG
OAM124	GTCGCACTGGCTGTTACTTC
OAM125	CACATGACCAGGAGCTTCGT
OAM139	TCGAGGGTCATTTTGCAAAAGTTGTTGACTTGAACAAACGTTTGATTCATAATGTGTGTA
OAM140	AGCTTACACACATTATGAATCAAACGTTTGTTCAAGTCAACAACTTTTGCAAAATGACCC
OAM148	GCATGCATGCGTAACACACAGGAAACAGCTATGAAAGTAAGCACCAAAGACAAAATTA
OAM149	GCATGGATCCGAACCGCTACCGTTGGTGAGCGCCACGTCT
OAM165	ACCGAATTAGCTTGCATGCGGCTAGCTCTAGTTGGTGAGCGCCAC
OAM166	ACCGAATTAGCTTGCATGCGGCTAGCTCTA
OAM200	CAATGAATGATCTGGCTGTGAG
OAM201	GCTTACTTTCATACGGCTCACTC
OAM202	TAGTATCAAGAGGAAGGAGTGAGCCGTATGAAAGTAAGCACCAAAGACAA
OAM203	TATCTAGAGGGAAACCGTTGTGGTCTAGTTGGTGAGCGCCAC
OAM204	AGGAGGAACTATATCCGGATCTGGACCAACTAGCACCGTTCCAA
OAM205	TTCAAGGCTGTCATAAAGCTC
OEB009	ATCAGCGCTCTGGTGATTG
OEB010	TTTTGCACAGCCTTAGCTTC
OEB024	ATACATATGAAAGTAAGCACCAAAGACA
OEB025	CGTTTTGAACAAACGTTTGATTAA
OEB026	TTAATCAAACGTTTGTTCAAAACG
OEB041	TATGGCTAGCATGAAAGTAAGCACCAAAGACA
OEB042	GGTGCTCGAGGTTGGTGAGCGCCACGTCTC
OEB092	Biotin-GCCTTTTCGTTTTGAACAAACGTTTGATTAAAACAAATAGC
OEB093	GCTATTTGTTTTAATCAAACGTTTGTTCAAAACGAAAAGGC
OJH001	CATATGTAAGATTTAAATGCAACCG
OJH002	CTACAAGGTGTGGCATAATGTGT
OJH133	GCAGGAATTCGACTCTCTAGCTTGAGG
OJH179	CCAGATCCGGATATAGTTCCTCCT
OJH180	ACCACAACGGTTTCCCTCTAGATA
OJH185	CAGGAATTCGACTCTCTAGC

OJH186	CTCAGCTAGCTAACTCACATTAATTGCGTTGC
OJW167	GCATGCATGCGTAACACACAGGAAACAGCTATGAAAGTAAGCACCAAAGACAAAATTA
OJW168	GCATGGATCCGAACCGCTACCGTTGGTGAGCGCCACGTCTCCT
OJW169	GCATGCATGCGTAACACACAGGAAACAGCTATGTTGGAGTTCGAAACAAACATAGAC
OJW170	GCATGGATCCGAACCGCTACCGCCGCGTTTATTACGGTTTCTTAAGA
OJW171	GCATGGATCCGGGCAGCGGTATGAAAGTAAGCACCAAAGACAAAATTA
OJW172	GCATGGATCCCTAGTTGGTGAGCGCCACGTC
OJW173	GCATGGATCCGGGCAGCGGTATGTTGGAGTTCGAAACAAACATAGAC
OJW174	GCATGGATCCTTAGCCGCGTTTATTACGGTTTCTTAA
OLM048	GCCGCTAGCATGAAAGTAAGCACCAAAGAC
OLM049	GCGCTCGAGGTTGGTGAGCGCCACGTC
OYD070	GTGTGGAATTGTGAGCGGATAAC

Table S4. Polymorphisms identified in the rLOF selection-screen

BAM	SNP	AA	SNP	AA	SNP	AA
**449**	**157 G->A**	**E53K**				
**462**	**181 G->A**	**E61K**				
**409**	**304 C->A**	**R102S**				
**407**	**304 C->T**	**R102C**				
410	304 C->T	R102C				
412	304 C->T	R102C				
442	304 C->T	R102C				
405	340 T->A	L114I				
402	346 A->T	R116W				
**440**	**346 A->T**	**R116W**				
**443**	**348 G->C**	**R116S**				
**403**	**350 A->G**	**E117G**				
441	350 A->G	E117G				
**444**	**351 A->T**	**E117D**				
463	351 A->T	E117D				
406	368 T->C	L123P				
**411**	**458 T->G**	**L153R**				
**400**	**535 G->A**	**E179K**				
459	535 G->A	E179K				
537	105 T->C	D35D	346 A->T	R116W		
404	145 G->C	G49R	348 G->C	R116S		
538	147 A->G	G49G	193 A->G	K65E	351 A->T	E117D
401	157 G->A	E53K	198 G->A	T66T	444 G->A	L148L
460	216 C->T	S72S	414 A->T	E138D	535 G->A	E179K

447	267 T->A	D89E	347 G->C	R116T	438 G->A	L146L

Table S5. Data collection, phasing and refinement statistics for the RefZ structure

**PDB ID**	6MJ1
**Data collection**	
Space group	$P\ 4_1\ 2_1\ 2$
Cell dimensions	
a, *b*, *c* (Å)	100.021, 100.021, 100.177
α, *β*, *γ* (°)	90, 90, 90
Resolution (Å)	2.6
R_{merge}	0.11 (0.79)
$I / \sigma I$	11.59
Completeness (%)	100 (100)
Redundancy	17.6 (15.6)
**Refinement**	
Resolution (Å)	44.952-2.6
No. reflections	16,039
R_{work} / R_{free}	22.20 / 25.36
No. atoms	
Protein	1,683
Water	25
B-factors	
Protein	76
r.m.s. deviations	
Bond lengths (Å)	0.009
Bond angles (º)	1.082

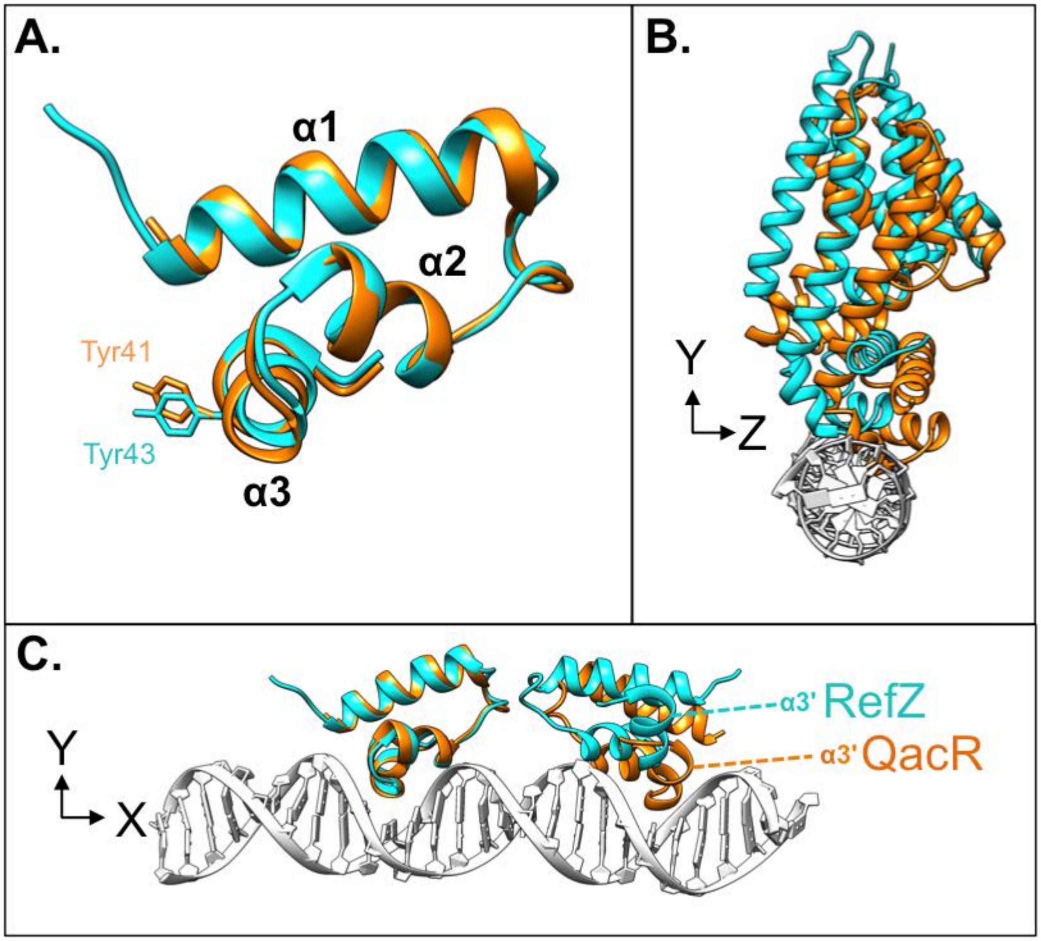

Figure S1. Superimposition of the N-terminal domains of RefZ and QacR. (A) Superimposition of the HTH domains of RefZ (cyan) and QacR (orange)(PDB: 1JT6)(4). The Y43 residue on α3 of RefZ, which is required for DNA binding and the corresponding residue in QacR (Y41) are shown as sticks. (B) Superimposition of RefZ dimer (cyan) with the QacR dimer (orange) bound to *IR1* DNA (white)(PDB: 1JT0)(5). (C) Superimposition of the HTH domains of RefZ (cyan) with QacR (orange) bound to *IR1* DNA (white)(PDB 1JT0).

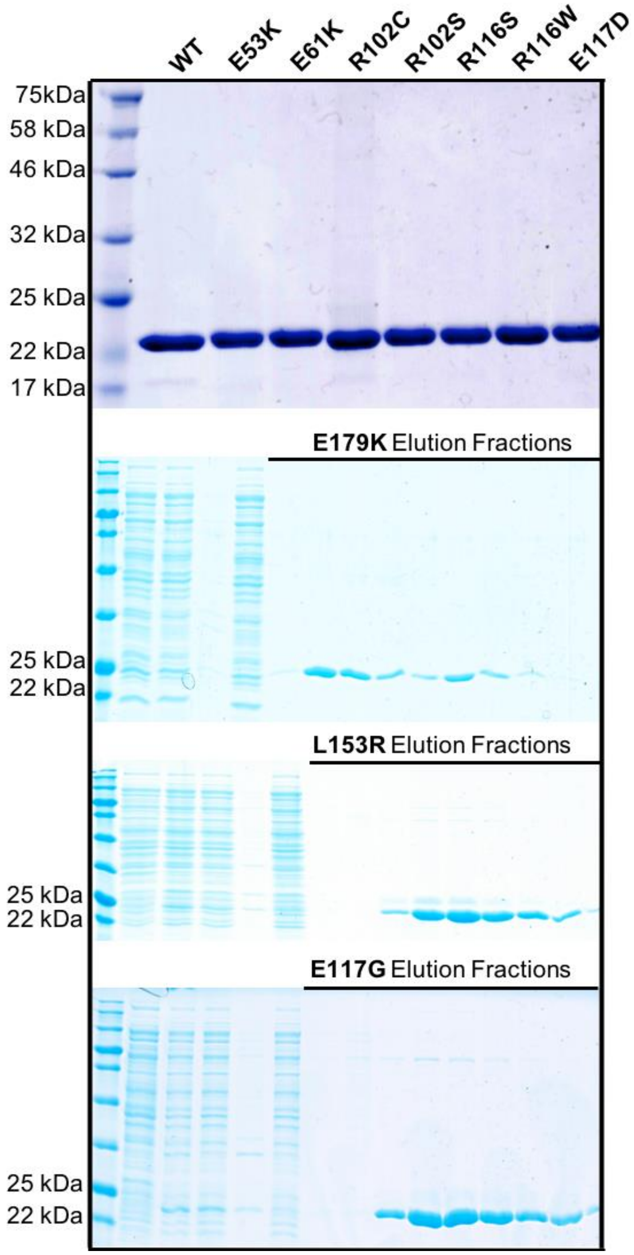

Figure S2. Example purification profiles of wild-type RefZ and rLOF variants. The top gel was loaded with 5 µg protein/lane and stained with coomassie blue dye (R-250). Gels below show example elution profiles from Nickel-NTA agarose beads. The elution gels were stained with coomassie brilliant blue dye (colloidal coomassie, G-250). G-250 is approximately 10 times more sensitive than R-250, allowing for detection of less abundant proteins.

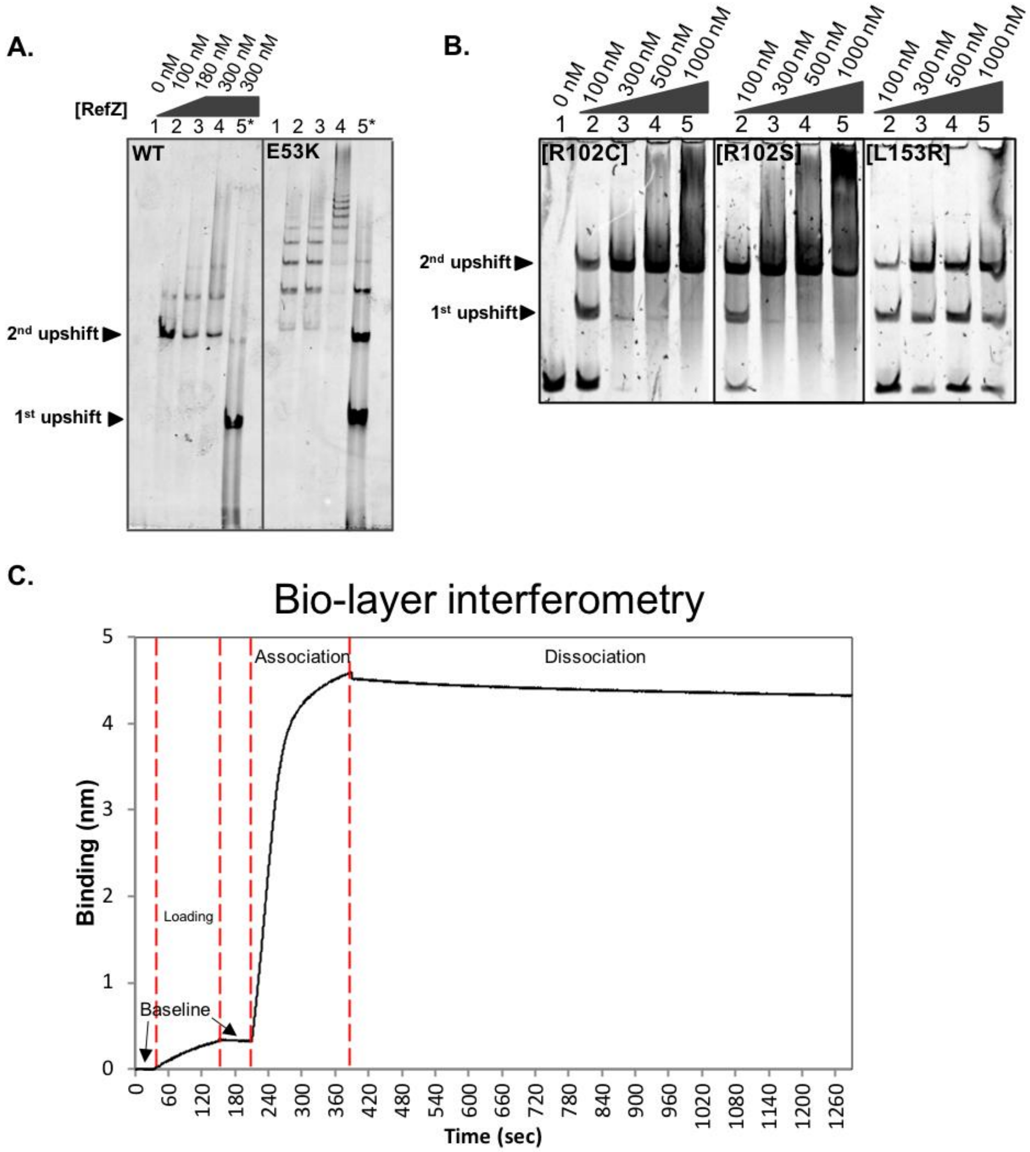

Figure S3. EMSA laddering behavior of wild-type RefZ and rLOF variants. (A) Laddering of DNA in the EMSAs can be observed for wild-type RefZ and to a greater extent E53K when samples are resolved at 200 V on a 7.5% TBE gel. (B)The rLOF variants R102C, R102S, and L153R do not exhibit laddering when samples are resolved at 200 V on a 7.5% TBE gel. (C) Typical bio-layer interferometry binding curve for wild-type RefZ with *RBM*-containing DNA. Sensors are pre-equilibrated for 10 min in DNA binding buffer (150 mM KCl and 10 mM Tris [pH 8]) at room temperature (not shown). The experiment is then initiated and performed at 30°C to establish a 30 sec baseline. The streptavidin sensor is dipped into a solution of biotinylated dsDNA (a 41 bp segment centered on RBM_{L1}) for 2 min. After incubation a new baseline is established by returning the biosensor to the DNA-binding buffer for 30 sec. The biosensor is then moved to a well containing 800 nM protein for 3 min to monitor association. The sensor is then transferred to a well containing fresh DNA-binding buffer to monitor dissociation for 15 min.

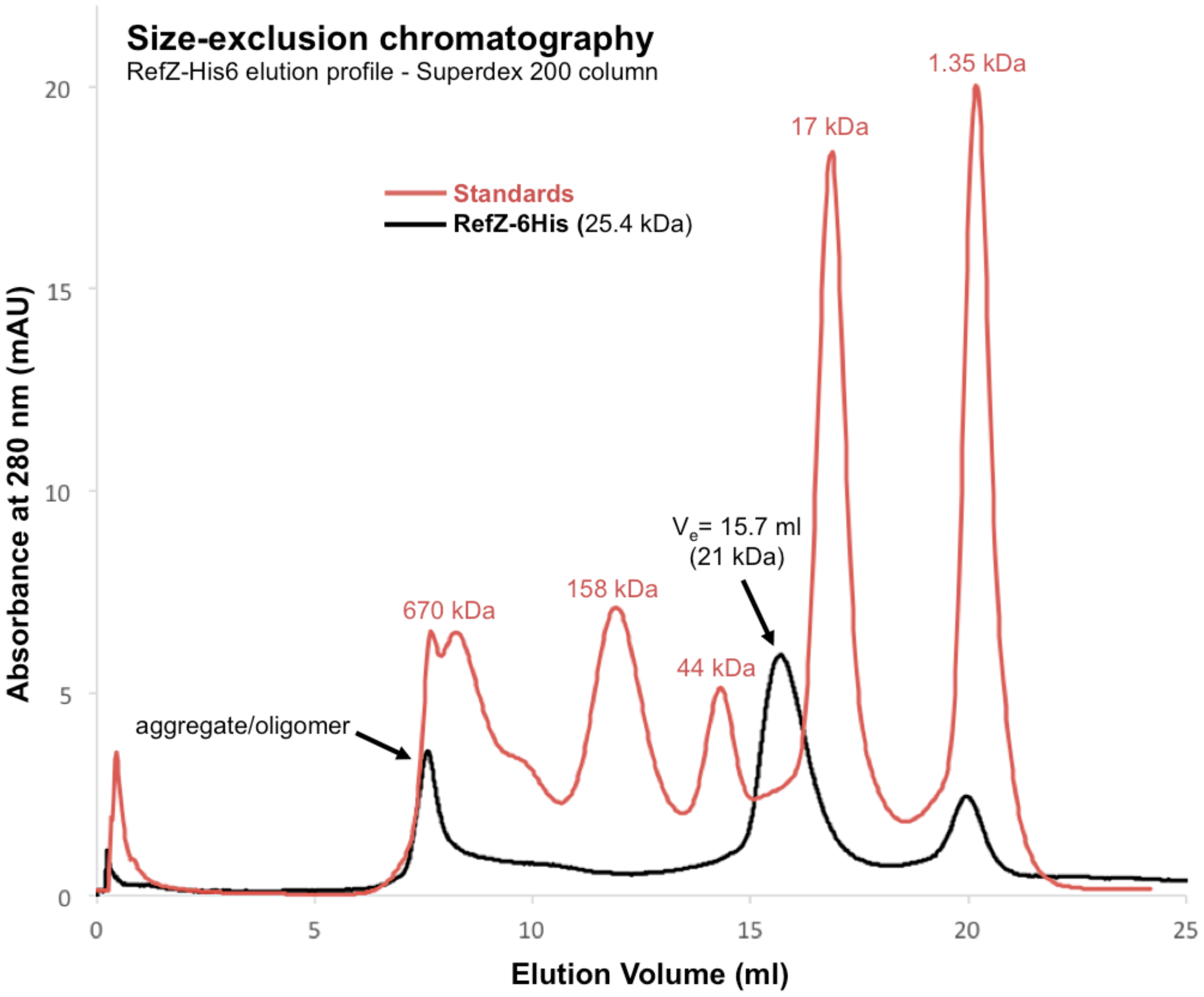

Figure S4. Size-exclusion chromatogram for WT RefZ. An example Superdex 200 elution profile for 200 µl of 1 µg ml^{-1} RefZ-His6 (7.7 nmol) ran with 50 mM Tris-HCl [pH 9], 300 mM KCl and 10% (v/v) glycerol. Absorbance at 280 nm is shown on the Y-axis (mAU – milliabsorbance units). Aggregated RefZ elutes at 7.6 ml, near the column void volume (7.0 ml)

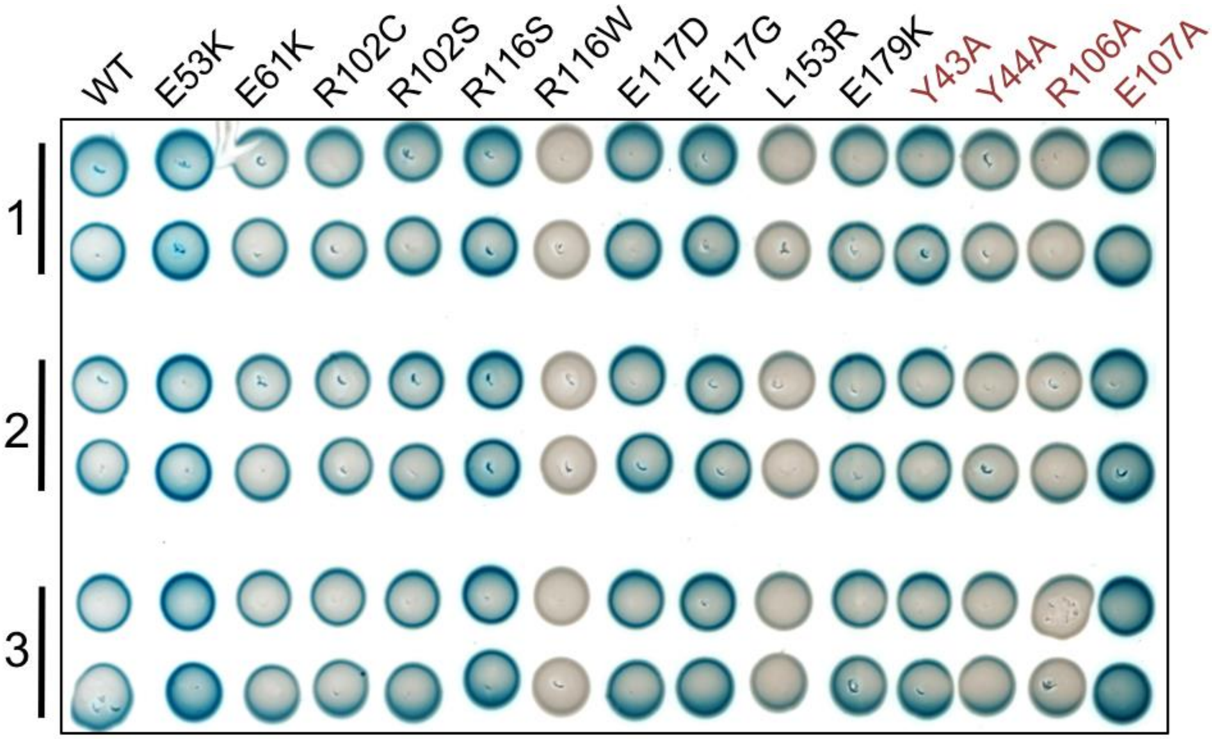

Figure S5. Bacterial two-hybrid assay spot plate replicas of RefZ and rLOF self-interaction. Culture from three biological independents were spotted in duplicate on M9 glucose minimal plates supplemented with 25 µg ml^{-1} kanamycin, 50 µg ml^{-1} ampicillin, and 40 µg ml^{-1} X-gal and grown for 44 h at room temperature. Strains are identical to those shown in Fig 7B.

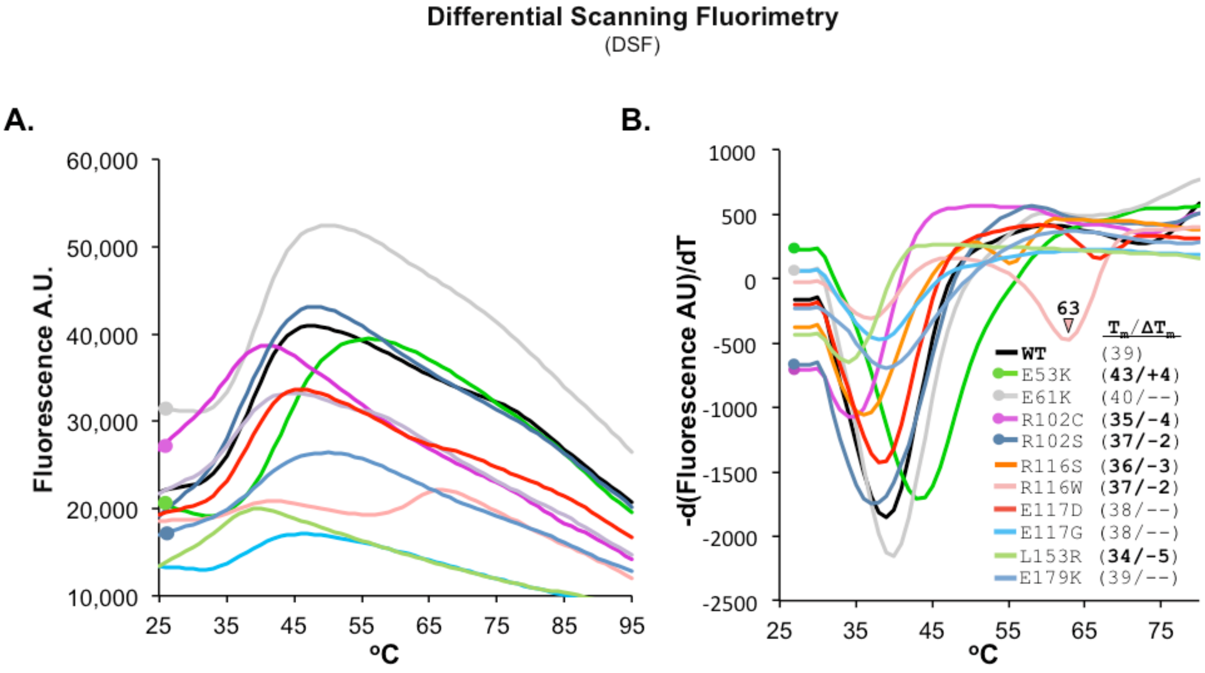

Figure S6. Thermostability of RefZ and the rLOF variants. DSF estimates of wild-type RefZ-His6 and rLOF-His6 variant stability reported by fluorescence of SYPRO orange as a function of increasing temperature. (A) Representative sigmoidal melting curves. (B) T_m values were calculated by determining the temperature at which the first derivative d(RFU)/dT is at a minimum. ΔT_m is the difference between the wildtype RefZ and each rLOF variant. Differences less than 1.5^{o}C were not considered to be significant and are shown as dashes.

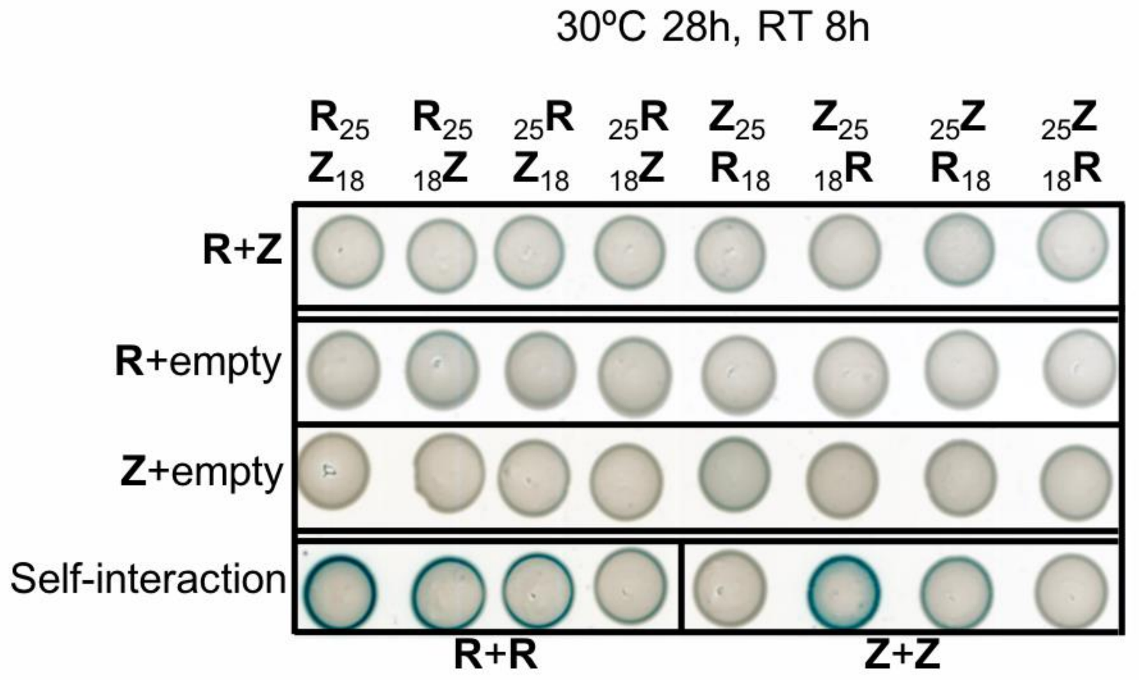

Figure S7. Bacterial two-hybrid assay for RefZ and FtsZ. *E. coli* DHP1 (*cya*-) co-transformants containing plasmids harboring N-terminal or C-terminal fusions of RefZ and FtsZ to T25 and T18 tags were grown in Lysogeny broth at 37° C in the presence of 25 µg ml^{-1} kanamycin, 50 µg ml^{-1} ampicillin, and 0.1% glucose as described in Methods. Cultures were normalized to the lowest OD_{600} reading and 8 µl were spotted on M9 glucose minimal plates supplemented with 25 µg ml^{-1} kanamycin, 50 µg ml^{-1} ampicillin, and 40 µg ml^{-1} X-gal and grown for 28 h at 30°C, followed by 8 h at room temperature.

REFERENCES

1. **Gibson DG, Young L, Chuang RY, Venter JC, Hutchison CA, 3rd, Smith HO.** 2009. Enzymatic assembly of DNA molecules up to several hundred kilobases. Nat Methods **6:**343-345.
2. **Wagner-Herman JK, Bernard R, Dunne R, Bisson-Filho AW, Kumar K, Nguyen T, Mulcahy L, Koullias J, Gueiros-Filho FJ, Rudner DZ.** 2012. RefZ facilitates the switch from medial to polar division during spore formation in *Bacillus subtilis*. J Bacteriol **194:**4608-4618.
3. **Miller AK, Brown EE, Mercado BT, Herman JK.** 2016. A DNA-binding protein defines the precise region of chromosome capture during *Bacillus* sporulation. Mol Microbiol **99:**111-122.
4. **Schumacher MA, Miller MC, Grkovic S, Brown MH, Skurray RA, Brennan RG.** 2001. Structural mechanisms of QacR induction and multidrug recognition. Science **294:**2158-2163.
5. **Schumacher MA, Miller MC, Grkovic S, Brown MH, Skurray RA, Brennan RG.** 2002. Structural basis for cooperative DNA binding by two dimers of the multidrug-binding protein QacR. EMBO J **21:**1210-1218.
